# Feasibility of ending tuberculosis in Shangrao City through active intervention measures: a mathematical study

**DOI:** 10.3389/fpubh.2025.1614339

**Published:** 2025-09-10

**Authors:** Mingshu Xu, Yue He, Qiao Liu, Qiuping Chen, Zeyu Zhao, Zheng Xu, Chongfei Shu, Jun Xia, Yuyan Yang, Laurent Gavotte, Roger Frutos, Huiming Ye, Yanhua Su, Xiaolan Wang, Zhen Liu

**Affiliations:** 1 Shangrao Centre for Disease Control and Prevention, Shangrao City, China; 2 State Key Laboratory of Vaccines for Infectious Disease, Xiang An Biomedicine Laboratory, State Key Laboratory of Molecular Vaccinology and Molecular Diagnostics, National Innovation Platform for Industry-Education Intergration in Vaccine Research, School of Public Health, Xiamen University, Xiamen City, China; 3 CIRAD, URM 17, Intertryp, Montpellier, France; 4 Université de Montpellier, Montpellier, France; 5 Espace-Dev, Université de Montpellier, Montpellier, France; 6 Department of Laboratory Medicine, Fujian Key Clinical Specialty of Laboratory Medicine, Women and Children’s Hospital, School of Medicine, Xiamen University, Xiamen City, China; 7 Shangrao People’s Hospital, Shangrao City, China

**Keywords:** tuberculosis, dynamic model, end tuberculosis, active case finding, tuberculosis preventive therapy

## Abstract

**Objective:**

China faces significant challenges in ending tuberculosis (TB). Active case finding (ACF) and TB preventive therapy (TPT) have proven to be critical measures in reducing TB incidence. This study uses a transmission dynamics model to identify the optimal intervention strategies for achieving WHO’s TB elimination targets in Shangrao City. The findings guide targeted TB control efforts in similar settings.

**Methods:**

To account for COVID-19 pandemic disruptions, we first used a seasonal autoregressive integrated moving average (SARIMA) model to predict and substitute the reported TB incidence during 2020–2023. Subsequently, we developed an age-stratified dynamic transmission model using surveillance data from Shangrao City’s Infectious Diseases Reporting System (IDRS) between 2008 and 2023 to evaluate tuberculosis transmission patterns across age groups. The model assessed the effectiveness of key interventions including active case finding (ACF), latent tuberculosis infection (LTBI) screening, and tuberculosis preventive treatment (TPT).

**Results:**

The model fit well with the reported data (R^2^ = 0.53, *p* < 0.001). Preventive treatment measures can fully achieve the goal of reducing incidence. All five TPT regimens showed potential to meet the TB elimination targets, with the 3HP regimen (weekly rifapentine + isoniazid for 3 months) performing the best. With the proportion of post-detection consent to TPT of 0.6 and rate of LTBI screening of 0.5, the 3HP regimen met the 2030 and 2035 incidence targets, with projected rates of 15.27/100,000 and 7.98/100,000, respectively.

**Conclusion:**

The current TB control efforts face significant challenges, with a considerable gap remaining in achieving TB elimination targets. Combining ACF with TPT presents a promising strategy to reach these goals. Older tuberculosis (TB) patients constitute a high-risk population, and effective prevention and treatment in this group are critical to achieving future TB elimination goals. To reduce the risk of recurrence and reinfection, enhanced follow-up monitoring of older patients should be prioritised alongside targeted health education interventions tailored to high-risk groups.

## Introduction

Directly Observed Treatment Short-Course (DOTS) has been promoted globally by the World Health Organization (WHO) since 1993. China has actively advanced tuberculosis (TB) prevention and control efforts, achieving full coverage of the DOTS strategy around 2004, which facilitated standardized TB treatment. Despite significant progress in TB control, substantial gaps remain (1). In 2015, WHO proposed the End TB Strategy, setting milestone targets every five years starting from 2015 (2). The ultimate goals include reducing the TB incidence rate by 80% and the TB mortality rate by 90% by 2030 and achieving similar reductions by 2035 compared to 2015 levels. However, China’s rate of decline in TB incidence and mortality is concerning. Between 2015 and 2020, China’s TB incidence rate decreased by only 11% and the mortality rate decreased by just 13%, far below the expected targets by 2020 (3).

For decades, the fundamental strategy for TB control has primarily involved providing diagnosis and treatment through health service institutions. If the ultimate goal is to interrupt transmission, early and accurate case detection, rapid initiation and adherence to treatment, and preventive therapy for latent TB infection (LTBI) are crucial. Currently, relying on passive case finding and screening at health facilities is insufficient to effectively reduce TB incidence. Many countries have begun to implement community-based active case finding (ACF) on a large scale (4–7). However, the effectiveness of ACF implementation varies across different countries and regions and over different periods (8, 9). The goal of ACF is early detection of TB patients, reducing delays in diagnosis and treatment, and slowing the spread of the disease within communities. A review by Burke et al. indicated that when community-based ACF achieves sufficient intensity and coverage, it can significantly reduce TB prevalence in communities (4). Additionally, Marks et al. also demonstrated through cluster randomized trials that ACF significantly reduces TB prevalence among children (with microbiologically confirmed TB prevalence of 0.56 in the intervention group compared to the control group) (10). Despite the widespread implementation of ACF interventions globally, evidence of their effectiveness and the optimal methods for providing these interventions remain uncertain. The effectiveness of ACF may be closely related to specific circumstances and varies with factors such as TB prevalence, environment, healthcare accessibility, and socioeconomic conditions (11, 12). Identifying and treating active TB patients is currently the core measure of TB control strategies. However, further research is needed to determine the optimal combination of interventions.

In high-burden countries, many TB patients do not exhibit obvious symptoms, making it insufficient to wait for patients’ conditions to worsen before they seek treatment, which does not significantly reduce the transmission and incidence rates (13). In 2018, the United Nations High-Level Meeting identified LTBI screening and TB preventive therapy (TPT) as key indicators for global TB control. Adequate diagnosis and therapy of LTBI are crucial factors in efforts to stop TB epidemics. Romain et al. (14) research emphasized the importance of early identification of active TB patients and screening and treating LTBI patients. The goal of LTBI management is to prevent latent infections from progressing to active TB and to reduce the reservoir of future TB cases. Randomized controlled trials have shown that TPT is effective in preventing disease progression (15). However, the effectiveness of LTBI therapy based on different TPT regimens varies greatly, with protection rates ranging from 0 to 61%, completion rates from 43 to 90%, and adverse reaction rates from 0 to 10%. Currently, systematic and comprehensive LTBI screening and TPT for high-risk groups have not been fully implemented in China, resulting in a lack of comprehensive data on LTBI screening and TPT.

Empirical studies cannot fully determine which interventions and technologies should be used, as it is challenging to test all possible approaches on a large scale before policy decisions are made (16). Mathematical models, however, can transform existing knowledge and assumptions—such as local epidemiology, scale-up strategies, and intervention effectiveness—into predictive outcomes and estimate uncertainties (17, 18). Transmission dynamics models can effectively simulate the impact of TB interventions and quantitatively evaluate their implementation outcomes.

Shangrao City has long prioritized TB prevention and control, making significant progress and achieving the goals of the 12th Five-Year TB Control Plan. However, current TB control efforts still face considerable challenges. In 2016, the reported incidence rate of TB in Shangrao City was 73.64 per 100,000, ranking third among all Class B infectious diseases in the city (19). The current prevention and control service system cannot fully meet the needs under the new situation. Therefore, to determine the effectiveness of TPT and ACF in ending TB epidemics, we chose Shangrao City as the study area. With its unique geographical conditions, TB control status, and extensive control practices, Shangrao City serves as a representative example. Exploring interventions that can help Shangrao City end its TB epidemic could provide significant insights for other similar cities nationwide. Therefore, this study first employed a SARIMA model to reconstruct normative TB incidence trends by replacing the observed 2020–2023 data, then established an age-stratified transmission dynamic model to evaluate the feasibility of reaching the WHO End TB Strategy target in Shangrao City. The model systematically assessed the combined effectiveness of active case finding (ACF), latent tuberculosis infection (LTBI) screening, and tuberculosis preventive treatment (TPT).

## Methods

### Study design and area

Shangrao City is located in the northeastern part of Jiangxi Province, adjacent to Zhejiang, Anhui, and Fujian provinces. It covers a total area of 22,737 square kilometers. Shangrao City currently administers over 12 prefecture-level cities (20, 21). By the end of 2023, the city had a resident population of 6,395,947, with an urbanisation rate of 57.06% (22).

In this study, based on the natural history of TB and TB incidence data from Shangrao City, we employed an *SL*_A_*L*_B_*IDR* model structured by age to quantify the transmission dynamics across different age groups. Building on this framework, we assessed the impact of proactive interventions on reducing TB incidence, including ACF, LTBI screening, and TPT. Additionally, we delved into the differential effects of chemoprophylaxis and immunotherapy within TPT regimens. These research findings aim to provide scientific rationale and implementation guidance for Shangrao City’s goal of achieving TB elimination.

### Data collection

We collected TB data from the Infectious Diseases Reporting System (IDRS) of the Shangrao Center for Disease Control and Prevention from 2008 to 2023, including information such as diagnosis date, symptom onset date, first consultation date, treatment outcomes, start and end of treatment dates, and age. Additionally, we obtained annual population data segmented by age from the Shangrao Statistics Bureau for 2008 to 2023 (23). All parameters with definitions, values, and sources are listed in Supplementary Table S1.

### Definitions and classification

Diagnostic delays in TB are categorised into patient and healthcare system delays. Patient delay refers to the time interval between the patient’s self-recognition of tuberculosis-related symptoms and their first presentation to any healthcare facility (24). Healthcare system delay refers to the period from this initial healthcare contact until definitive laboratory-confirmed TB diagnosis. This definition explicitly applies to symptomatic cases, as asymptomatic individuals identified through screening programs do not exhibit patient delay by epidemiological convention. Patient diagnosis delay was calculated by subtracting the diagnosis date from the symptom onset date, and healthcare system diagnosis delay was determined by subtracting the treatment initiation date from the diagnosis date.

Pulmonary tuberculosis refers to TB lesions occurring in the lung tissue, trachea, bronchi, and pleura, including pulmonary parenchyma tuberculosis, tracheobronchial tuberculosis, and tuberculous pleurisy. Diagnosis is primarily based on a combination of bacteriological examination, clinical symptoms, chest imaging, and epidemiological history, with laboratory tests focusing on sputum smears and cultures as the key methods (25).

Since 2019, the classification criteria for pulmonary tuberculosis have been adjusted to “rifampicin-resistant, bacteriologically positive, bacteriologically negative, and no bacteriological results.” Main age categories for parameters were children (0–14 years), adolescents and (15–64 years), and people aged 65 years or older (26). Given the elevated disease burden and unique clinical challenges of tuberculosis in older populations—particularly within China’s rapidly aging demographic—this study stratified cases into two critical cohorts: 15–64 years and ≥65 years. All data were reclassified according to the latest diagnostic standards (27), excluding cases of extrapulmonary TB and nontuberculous mycobacteria (NTM), which accounted for 484 cases or 0.65% of the total (484/74,948). Given that children primarily present with primary pulmonary tuberculosis and have a lesser impact on disease transmission (26, 28, 29), this study also excluded the 0–14 age group, amounting to 423 cases or 0.56% of the total (423/74,948). Ultimately, 74, 040 patients were included in this analysis.

### Data handling

From 2020 to 2023, the incidence of tuberculosis in Shangrao City was affected by the prevention and control of the novel coronavirus and decreased to a certain extent. To exclude this effect, this study used the SARIMA model to predict the incidence of pulmonary TB from 2020 to 2023 based on incidence data from 2008 to 2019, and the predicted data for these four years were used to replace the actual data. The SARIMA model is a statistical model for time-series prediction that models and predicts data by combining autoregressive (AR), difference (I), and sliding averages (MA). The autoregressive part describes the relationship between the current and past values, the difference part is used to remove trend or seasonal effects, and the sliding average part considers historical data of the error term. The SARIMA model is often used to predict time series data with historical dependencies and long-term trends (30), and in this study, it can effectively capture pulmonary TB onset trends and predict pulmonary TB onset data after interference of the COVID-19 outbreak. However, the SARIMA model does not model the propagation process, and therefore cannot simulate the effects of some intervention measures (such as ACF, TPT, etc.).

### Model establishment

Our model considers multiple stages of TB progression, including self-clearance, early progression, late progression, immune stability, relapse, treatment, and diagnostic delays, forming a transmission dynamics model. Additionally, we accounted for differences in disease progression among different age groups by establishing an age-structured *SL*_A_*L*_B_*IDR* model. In this model, *S* represents the susceptible population, *L*_A_ represents early latent infections, *L*_B_ represents late latent infections, *I* represents active TB cases, *D* represents diagnosed, but untreated TB cases, and *R* represents recovered individuals. The model includes two age groups: 15–64 years and 65 years and older, denoted by subscripts *i* and *j*, respectively.

This model categorises the population into distinct compartments, each represented by a differential equation, to describe the dynamics of TB infection and recovery. The susceptible compartment (*S*) represents individuals who have not been exposed to *Mycobacterium tuberculosis* (*M. tuberculosis*) or those who have cleared the infection through a robust immune response governed by Equations 1, 7. The exposed compartment (*E*), also referred to as the LTBI group, included individuals who had been exposed to *M. tuberculosis* through contact with infected individuals. These individuals carry the bacteria but remain temporarily non-infectious. To capture progression within the latent stage, the exposed population was further divided into early latent infections (*L*_A_) and late latent infections (*L*_B_), governed by Equations 2, 3, 8, 9. Infectious compartment (I) represents individuals with active TB disease, with the dynamics defined in Equations 4, 10. The model also incorporated a diagnosed but untreated compartment (*D*) (Equations 5, 11) to account for individuals identified as having TB but not yet undergoing treatment, highlighting a critical point in the treatment cascade. Finally, the recovered or removed compartment (*R*) includes individuals who have successfully completed treatment, whether cured or treatment-completed, and are thus non-infectious and asymptomatic. The dynamics are described by Equations 6, 12: This compartmental structure, defined by differential equations, enables a comprehensive representation of the TB transmission, progression, and recovery processes.

### Model assumptions

1. The model considers natural birth and death rates within the population.
2. Early Clearance (EC) is a self-protective mechanism by which the body rapidly clears *M. tuberculosis* before an adaptive immune response is generated, thereby preventing the progression to active TB. In this study, m represents the proportion of early clearance, and *θ* represents the rate of early clearance. Studies have shown that 30.2–58.2% of infected individuals experience early clearance within 8 weeks to 2 years, as confirmed by interferon-*γ* release assay (IGRA) testing (31). We also assume that TB patients have some capacity for spontaneous recovery, denoted by *φ*, the rate of spontaneous recovery.
3. Research indicates that 5–10% of latent TB infections progress to active TB (32), with variation in the rate of progression. Some individuals progress rapidly within the first two years after infection, particularly older individuals who face a higher risk of early progression. Others progress more slowly, with progression potentially taking more than 20 years.
4. Due to variations in immune status, therapy regimens, and adherence, recovered TB patients may relapse and become active TB cases again (33). This pathway from recovered TB patients to the LTBI (Latent Tuberculosis Infection) state is not considered in the modeling process (34).
5. The TB mortality risk is assumed to be the same for both untreated TB patients and those diagnosed but awaiting treatment.

**Figure 1:**
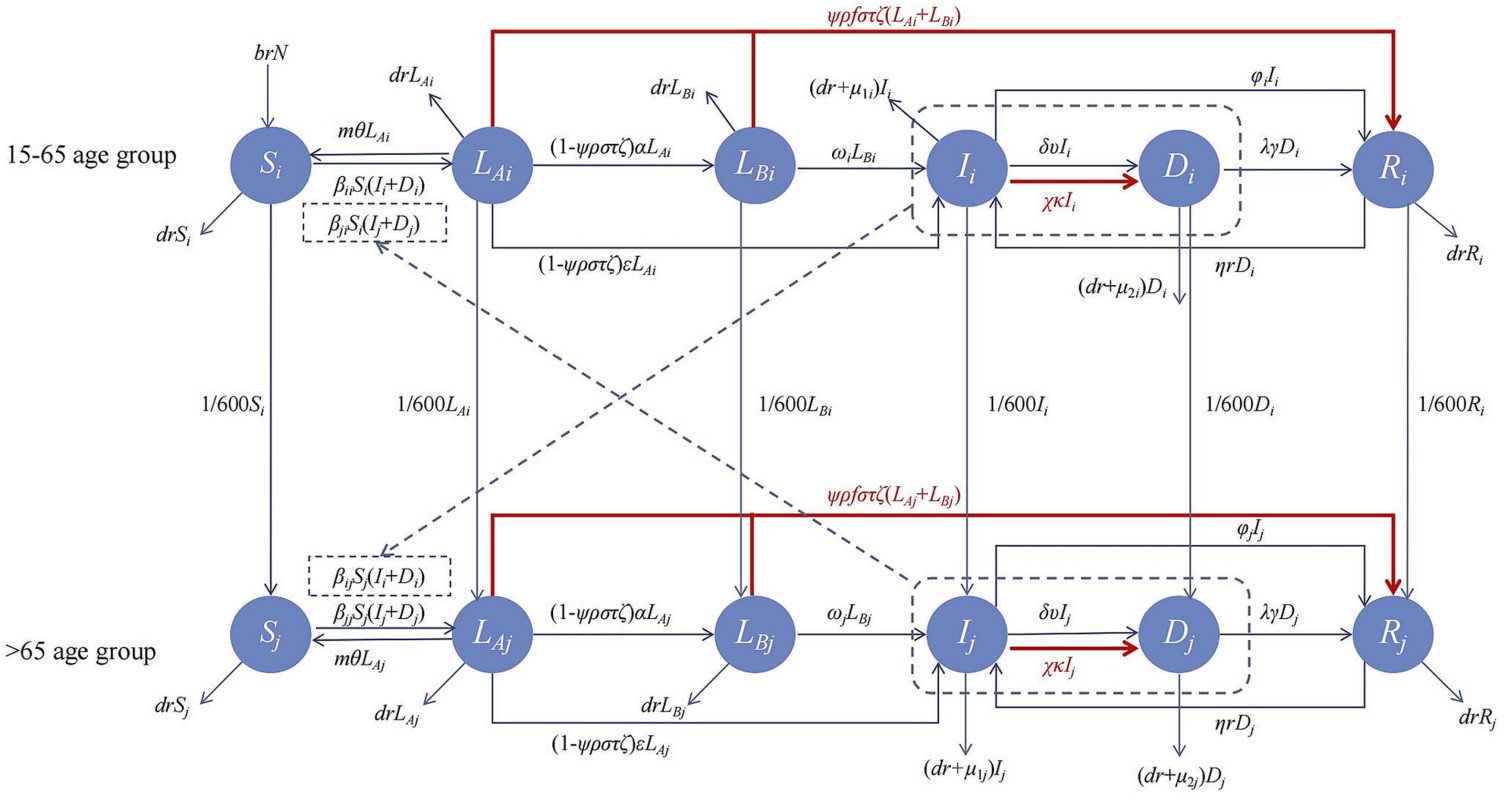
Model framework.

Figure 1 shows the *SL*_A_*L*_B_*IDR* model framework. The mathematical expressions for the differential equations in the *SL*_A_*L*_B_*IDR* model are as follows:


$$\begin{array}{l} \frac{{\mathrm{dS}}_{i}}{\mathrm{dt}}=\mathrm{brN}+\mathrm{m\theta}L_{\mathrm{Ai}}-\beta_{\mathrm{ii}}S_{i}(I_{i}+D_{i})-\beta_{\mathrm{ji}}S_{i}(I_{j}+D_{j})-\\ {\mathrm{drS}}_{i}-1/600S_{i} \end{array}$$ (1)

$$\begin{array}{l} \frac{dL_{\mathrm{Ai}}}{\mathrm{dt}}=-\mathrm{m\theta}L_{\mathrm{Ai}}+\beta_{\mathrm{ii}}S_{i}(I_{i}+D_{i})+\beta_{\mathrm{ji}}S_{i}(I_{j}+D_{j})-(1-\text{ψρστζ})\\ aL_{\mathrm{Ai}}-(1-\text{ψρστζ})\varepsilon L_{\mathrm{Ai}}-\text{ψρfστζ}L_{\mathrm{Ai}}-\\ \mathrm{dr}L_{\mathrm{Ai}}-1/600L_{\mathrm{Ai}} \end{array}$$ (2)

$$\begin{array}{l} \frac{dL_{\mathrm{Bi}}}{\mathrm{dt}}=(1-\text{ψρστζ})aL_{\mathrm{Ai}}-(1-\text{ψρστζ})\omega_{i}L_{\mathrm{Bi}}-\\ \text{ψρfστζ}L_{\mathrm{Bi}}-\mathrm{dr}L_{\mathrm{Bi}}-1/600L_{\mathrm{Bi}} \end{array}$$ (3)

$$\begin{array}{l} \frac{{\mathrm{dI}}_{i}}{\mathrm{dt}}=-(1-\text{ψρστζ})\omega_{i}L_{\mathrm{Bi}}+(1-\text{ψρστζ})\varepsilon L_{\mathrm{Ai}}+\\ r\eta_{i}R_{i}-\varphi_{i}I_{i}-{\mathrm{\delta \upsilon I}}_{i}-{\mathrm{\chi \kappa I}}_{i}-\mu_{1i}I_{i}-{\mathrm{drI}}_{i}-1/600I_{i} \end{array}$$ (4)

$$\frac{{\mathrm{dD}}_{i}}{\mathrm{dt}}={\mathrm{\delta \upsilon I}}_{i}+{\mathrm{\chi \kappa I}}_{i}-{\mathrm{\lambda \gamma D}}_{i}-\mu_{2i}D_{i}-{\mathrm{drD}}_{i}-1/600D_{i}$$ (5)

$$\begin{array}{l} \frac{{\mathrm{dR}}_{i}}{\mathrm{dt}}=\varphi_{i}I_{i}+{\mathrm{\lambda \gamma D}}_{i}-r\eta_{i}R_{i}+\text{ψρfστζ}\\ (L_{\mathrm{Ai}}+L_{\mathrm{Bi}})-{\mathrm{drR}}_{i}-1/600R_{i} \end{array}$$ (6)

$$\frac{{\mathrm{dS}}_{j}}{\mathrm{dt}}=\frac{1}{600S_{j}}+\mathrm{m\theta}L_{\mathrm{Aj}}-\beta_{\mathrm{jj}}S_{j}(I_{j}+D_{j})-\beta_{\mathrm{ij}}S_{j}(I_{i}+D_{i})-{\mathrm{drS}}_{j}$$ (7)

$$\begin{array}{l} \frac{dL_{\mathrm{Aj}}}{\mathrm{dt}}=\frac{1}{600L_{\mathrm{Aj}}}\pm \mathrm{m\theta}L_{\mathrm{Aj}}+\beta_{\mathrm{jj}}S_{j}(I_{j}+D_{j})+\beta_{\mathrm{ij}}S_{j}(I_{i}+D_{i})-\\ (1-\text{ψρστζ})aL_{\mathrm{Aj}}-(1-\text{ψρστζ})\varepsilon L_{\mathrm{Aj}}-\\ \text{ψρfστζ}L_{\mathrm{Aj}}-\mathrm{dr}L_{\mathrm{Aj}} \end{array}$$ (8)

$$\begin{array}{l} \frac{dL_{\mathrm{Bj}}}{\mathrm{dt}}=1/600L_{\mathrm{Bj}}+(1-\text{ψρστζ})aL_{\mathrm{Aj}}-(1-\text{ψρστζ})\\ \omega_{i}L_{\mathrm{Bj}}-\text{ψρfστζ}L_{\mathrm{Bj}}-\mathrm{dr}L_{\mathrm{Bj}} \end{array}$$ (9)

$$\begin{array}{l} \frac{{\mathrm{dI}}_{j}}{\mathrm{dt}}=1/600I_{j}-(1-\text{ψρστζ})\omega_{j}L_{\mathrm{Bj}}+(1-\text{ψρστζ})\varepsilon L_{\mathrm{Aj}}+\\ r\eta_{j}R_{j}-\varphi_{j}I_{j}-{\mathrm{\delta \upsilon I}}_{j}-{\mathrm{\chi \kappa I}}_{j}-\mu_{1j}I_{j}-{\mathrm{drI}}_{j} \end{array}$$ (10)

$$\frac{{\mathrm{dD}}_{j}}{\mathrm{dt}}=1/600D_{j}+{\mathrm{\delta \upsilon I}}_{j}+{\mathrm{\chi \kappa I}}_{j}-{\mathrm{\lambda \gamma D}}_{j}-\mu_{2j}D_{j}-{\mathrm{drD}}_{j}$$ (11)

$$\begin{array}{l} \frac{{\mathrm{dR}}_{j}}{\mathrm{dt}}=1/600R_{j}+\varphi_{j}I_{j}+{\mathrm{\lambda \gamma D}}_{j}-r\eta_{j}R_{j}+\\ \text{ψρfστζ}(L_{\mathrm{Aj}}+L_{\mathrm{Bj}})-{\mathrm{drR}}_{j} \end{array}$$ (12)

The parameter estimation of the model consisted of the following: rate of self-clearance, proportion of self-clearance, rate of early progression, rate of late progression, rate of immune stabilisation, and rate of spontaneous recovery, and other 11 parameter values, all of which were obtained from Ref. Based on IDRS data, we estimated the proportion of treatment success, time to treatment, and time to diagnostic delay. The population data were obtained from the Statistical Yearbook of the Shangrao City Bureau of Statistics. Details are presented in Supplementary Table S1. In this study, $\beta_{\mathrm{ii}}$ represents transmissibility from 15–65 age group to the 15–65 age group, $\beta_{\mathrm{ij}}$ represents transmissibility from the 15–65 age group to the ≥65 age group, $\beta_{\mathrm{ji}}$ represents transmissibility from the ≥65 age group to the 15–65 age group, and $\beta_{\mathrm{jj}}$ represents transmissibility from the ≥65 age group to the ≥65 age group.

To account for the seasonality effect on TB transmission, we incorporated a seasonality function into the model to fit the reported TB epidemic curve. The transmission rate $\beta_{t}$ is modelled as follows (Equation 13):


$$\beta_{t}=\beta_{0}(1+\sin(\frac{2\pi (t-c)}{T}))$$ (13)

where $\beta_{0}$ represents the baseline transmission rate (at *t* = 0), *c* is the time adjustment factor (months) aligning the seasonality curve with the observed data, and *T* denotes the seasonal cycle period, which enables the transmission rate to fluctuate periodically, effectively capturing the seasonal variation in TB incidence and aligning the model with observed trends from 2008 to 2023.

The parameter estimation employed maximum likelihood estimation (MLE) implemented through the bbmle package in R, minimizing the negative log-likelihood between modeled and observed monthly tuberculosis incidence data under Gaussian error assumptions. Optimization was conducted using the Nelder–Mead algorithm with rigorously defined convergence criteria, including gradient tolerance thresholds below 10^−4^ and function value tolerances under 10^−6^. Model validation incorporated comprehensive analytical approaches: visual trajectory inspection against empirical epidemic curves was complemented by residual diagnostics to verify error structure properties, while sensitivity analyses assessed parameter uncertainty bounds. Biological plausibility constraints were enforced throughout the estimation process to ensure epidemiologically meaningful solutions.

### Intervention simulation

Intervention 1: ACF of active cases, with a set screening frequency of one screening round per year. After each screening round, TB patients will enter the diagnosed waiting for treatment TB patient at a rate of *χκ*, where *χ* denotes the sensitivity of active screening and *κ* denotes the rate of ACF (35, 36). We simulated a change in the active screening rate between 30 and 100% to observe a decline in the number of cases. In Shangrao City, the current proportion of passive case detection is 74%, while active screening accounts for 30%. The rate of passive case detection was defined as the reciprocal of the average diagnosis delay, calculated annually based on the reported data. Diagnostic delay was defined as the interval between the recorded symptom onset and diagnosis dates.

Intervention 2: LTBI screening and TPT were implemented on top of the ACF; therefore, the LTBI screening rate was aligned with the ACF rate. We modeled the effect of LTBI screening and TPT by the proportion of post-detection consent to TPT *ρ* × rate of LTBI screening *σ*× rate of TPT *f* × LTBI diagnostic sensitivity *τ* × rate of TPT protection *ζ* × rate of TPT compliance *ψ*. It is assumed that a portion of early and late LTBI cases will be transferred to the recovered group R after each round of screening and TPT.

According to the Chinese tuberculosis preventive therapy guidelines, this study included five chemotherapeutic and immunoprophylactic treatment regimens. We assumed a consistent LTBI diagnostic sensitivity across all regimens. The differences in the intervention effects between the regimens were studied by comparing the protection rate, treatment duration, and compliance with the different regimens. The rate of TPT was defined as the reciprocal of treatment duration. To evaluate the impact of LTBI screening and TPT, we modelled changes in TB incidence based on varying levels of post-detection consent to TPT *ρ* (0.2–1.0) and LTBI screening coverage *σ* (0.3–1.0). The details of the regimens are provided in Table 1. The rate of TPT compliance for each therapy regimen was derived from relevant literature (37–39). Similarly, the LTBI diagnostic sensitivity was informed by referenced studies (40).

**Table 1:** Preventive treatment regimens.

Therapy regimens	Age range	Therapy duration	Rate of TPT protection	Rate of TPT compliance	Simulation
6H/9H (daily isoniazid for 6–9 months)	Adults and children	6–9 month	65–90%	50–60%	Changes in morbidity were modeled for different proportions *ρ* (0.2–1.0) of post-detection consent to TPT, and proportions *σ* (0.3–1.0) of LTBI screening, respectively.
3HP (weekly rifapentine + isoniazid for 3 months)	Adults and children over 5 years old	3 month	75%	90%	
3HR (daily rifampicin + isoniazid for 3 months)	Adults and children	3 month	68%	70%	
4R (daily rifampicin for 4 months)	Adults and children	4 month	68%	70% (39)	
Scheme 5: Prophylactic immunotherapy	15–65 years	2–3 month	54.7%	100% (40, 41)	

### Statistical analysis

Data analysis was performed using the R software (version 4.2.2, R). Model fitting was performed using the “deSolve” and “bbmle” packages in R version 4.2.2. Parameter estimation was performed using the Least Squares Method. Model fit was assessed using *R*-squared (*R^2^*) values and *p*-values to determine the explanatory power and statistical significance of the model. We used the “ggplot2” package in R version 4.2.2 to create graphical representations to visualise the findings and model predictions. The significance level for all statistical analyses was set at *p* < 0.05.

## Results

### SARIMA prediction

**Figure 2:**
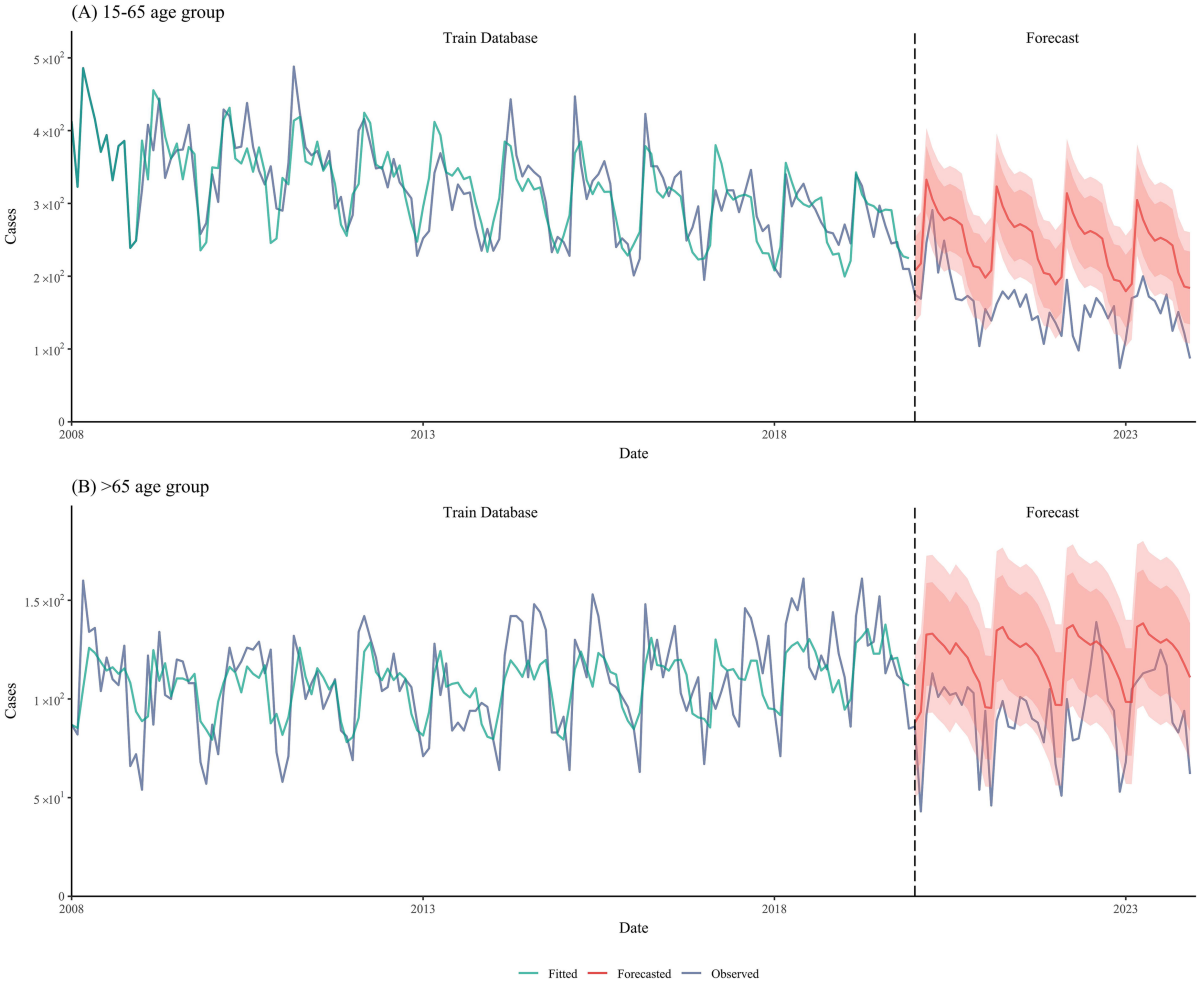
SARIMA model predictions of tuberculosis case incidence.

We employed SARIMA models to predict the incidence of tuberculosis in two age groups (15–65 and >65 years) from 2020 to 2023 based on the incidence data from 2008 to 2019. The SARIMA models for the two groups used different parameters: for the 15–65 age group, the forecast parameters were (0,0,0)(0,1,1)[12], whereas for the >65 age group, the forecast parameters were (1,1,1)(1,0,2)[12]. The prediction results were satisfactory, with R^2^ values of 0.70 and 0.68, respectively (Figure 2).

### Model fitting

We fitted a model to the tuberculosis incidence data from Shangrao City between 2008 and 2023. The results showed a good overall fit (R^2^ = 0.53, *p* < 0.001), indicating a significant relationship between the predicted and observed values. This difference was statistically significant (Supplementary Figure S1). The predicted results demonstrate distinct age-specific TB incidence trends: while the 15–65 age group showed a gradual decline before 2020, our adjusted projections (accounting for COVID-19 effects) suggest a potential slow increase during 2020–2035. In contrast, the >65 age group exhibits a consistent year-on-year increase in TB incidence throughout the study period.

### Intervention effects on ending TB

**Figure 3:**
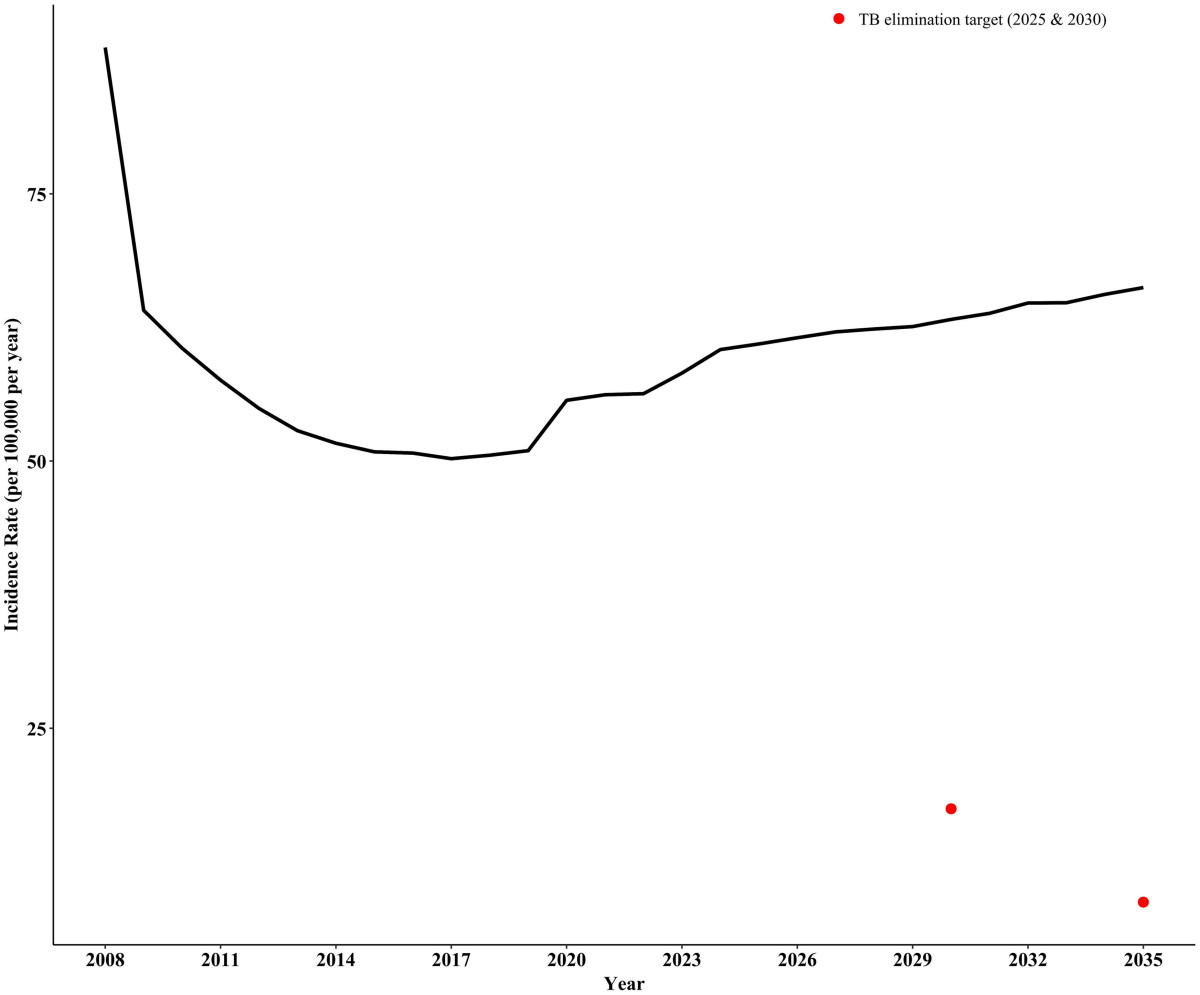
Prediction of incidence rate in Shangrao City without intervention.

Based on our predictions, without any intervention, continuing current control measures in Shangrao City will result in an estimated incidence rate of 62.03 per 100,000 in 2030 and 64.68 per 100,000 in 2035. These projections significantly exceed the target incidence rates for ending pulmonary tuberculosis (17.46 per 100,000 by 2030 and 8.73 per 100,000 by 2035) (Figure 3).

### Intervention a: ACF

**Figure 4:**
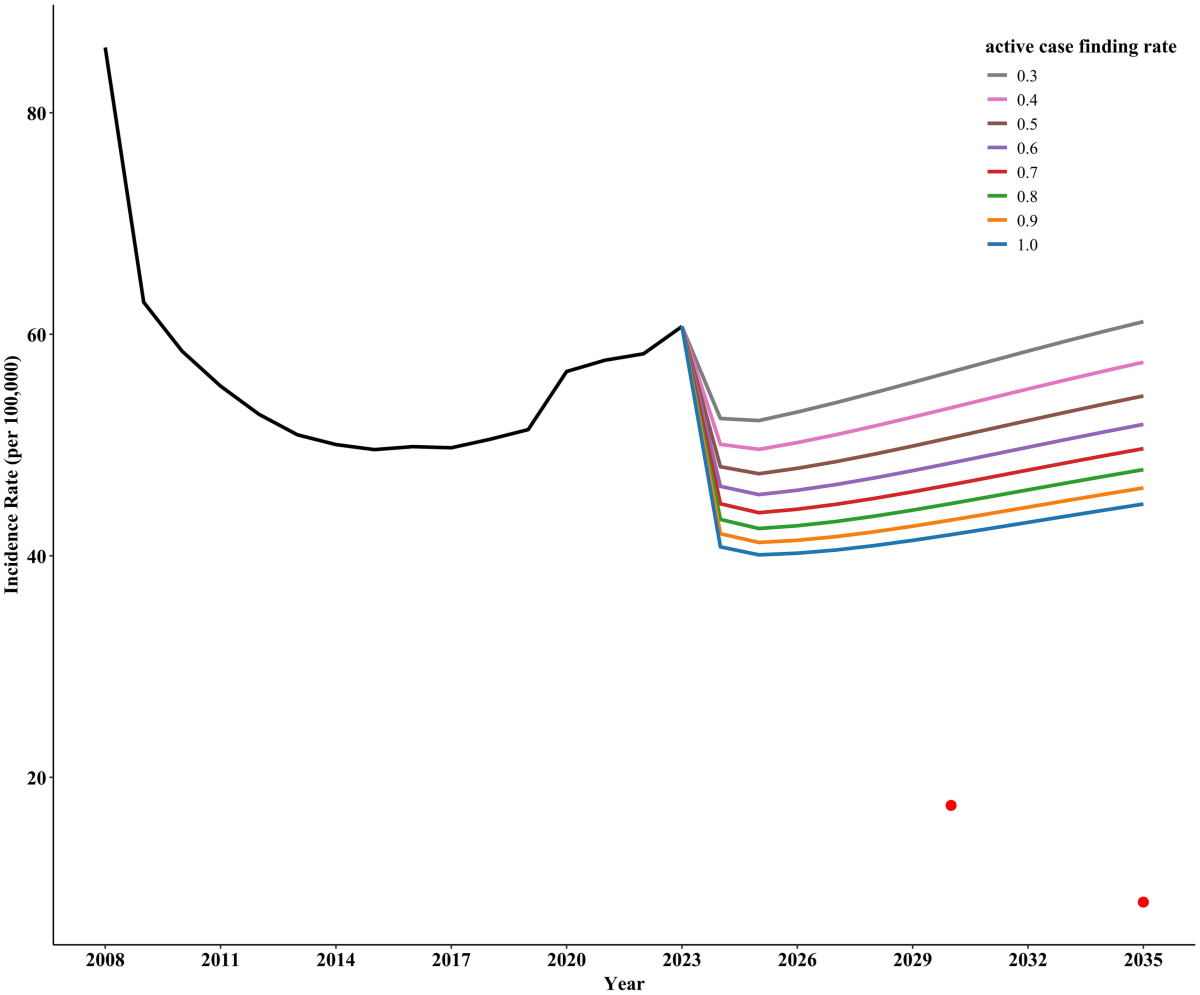
Prediction of incidence rate in Shangrao City based on Intervention A.

Implementing only ACF for active TB shows a gradual decline in incidence rates with increased screening intensity. However, even with a screening rate of 100%, Shangrao City would still not achieve the target incidence rate for ending pulmonary tuberculosis (Figure 4).

### Intervention B: LTBI screening and preventive therapy

**Figure 5:**
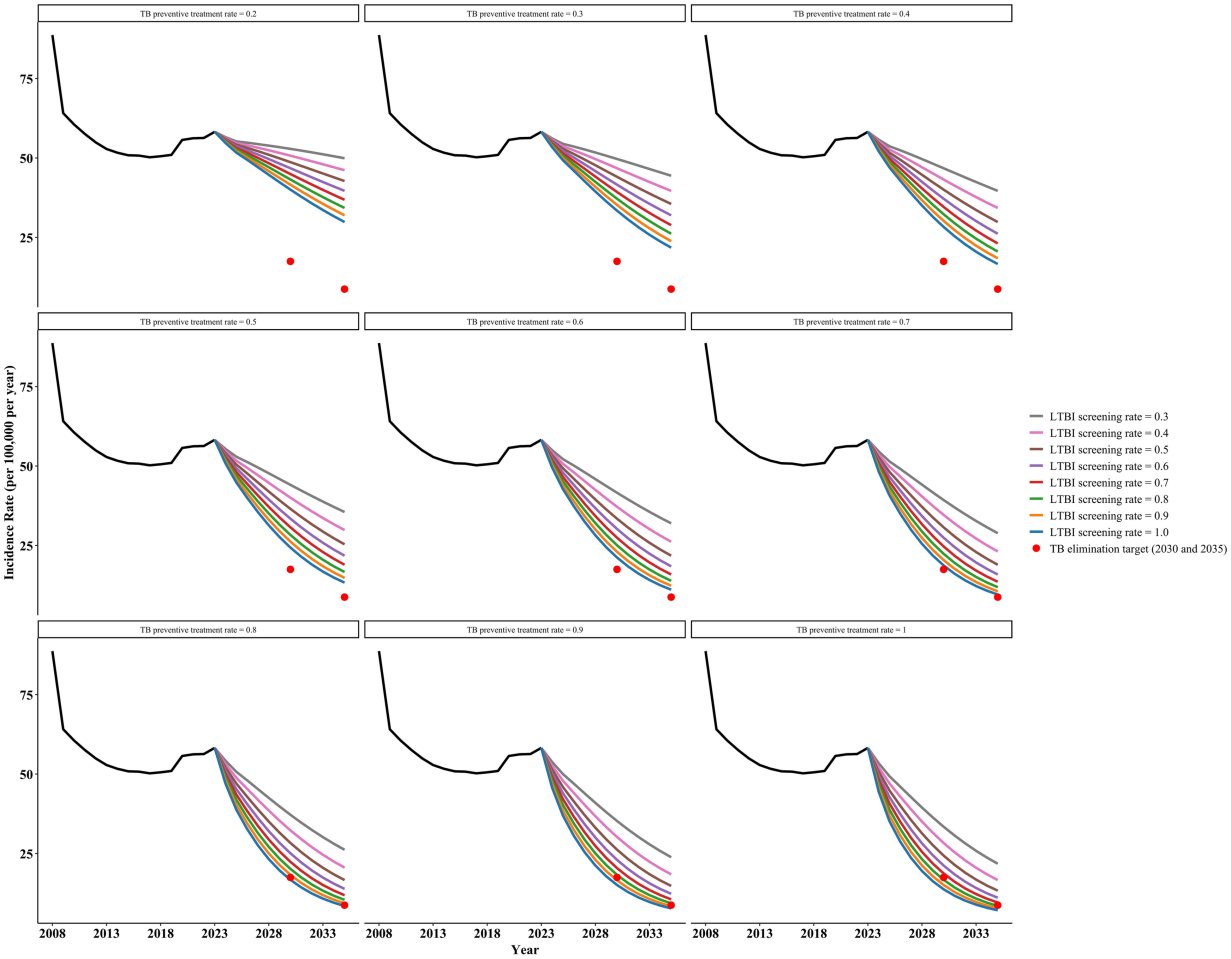
Prediction of incidence rate in Shangrao city based on intervention B scheme 1.

Under Intervention B, any preventive treatment regimen could reach the target incidence of ending TB by 2035, regardless of the regimen chosen (Figure 5 and Supplementary Figures S3–S6). The 3HP regimen (weekly rifapentine + isoniazid for 3 months) performed the best.

Scheme 1, with a post-detection consent to TPT rate of 0.9 and an LTBI screening rate of 0.9, can achieve the target incidence rates for 2030 and 2035, with projected incidence rates of 16.54 per 100,000 in 2030 and 8.40 per 100,000 in 2035. Scheme 2 (3HP) also achieves the corresponding target incidence rates when the post-detection consent rate is 0.5 and the LTBI screening rate is 0.5, with projected incidence rates of 17.06 per 100,000 in 2030 and 8.72 per 100,000 in 2035. Scheme 3 demonstrates superior effectiveness in reducing TB incidence compared to Scheme 4. Under Scheme 3 (with a TPT acceptance rate of 0.5 and LTBI screening rate of 0.8), the projected incidence rates would reach 15.84 and 8.20 per 100,000 population in 2030 and 2035, respectively. In contrast, Scheme 4 requires a higher LTBI screening rate of 0.9 (with an equivalent TPT acceptance rate of 0.5) to achieve comparable results (16.90 and 8.62 per 100,000 population in 2030 and 2035, respectively). Scheme 5, based on immunotherapy, slightly outperformed Scheme 3 when the consent and LTBI screening rates were equal, further reducing the incidence rates. The projected incidence rates for 2030 and 2035 are 14.65 per 100,000 and 7.79 per 100,000 people, respectively.

Furthermore, based on the above results, we found that increasing the proportion of post-detection consent to TPT while simultaneously reducing the LTBI screening rate can also achieve the target incidence rates for ending tuberculosis. As the proportion of post-detection consent for TPT increases, the required LTBI screening rate decreases accordingly. For example, in Scheme 2, when the consent rate increases to 0.9, an LTBI screening rate of 0.3 is sufficient to achieve the target incidence rates, with a projected incidence rate of 16.26 per 100,000 in 2030 and 8.38 per 100,000 in 2035. Increasing both parameters accelerates the achievement of target incidence rates for ending pulmonary tuberculosis. For instance, with a consent rate of 0.9 and an LTBI screening rate of 0.9, the target (incidence rate of 8.73 per 100,000) could be achieved as early as 2031.

## Discussion

This study, based on an age-structured transmission dynamics model, systematically evaluated the effects of ACF, LTBI screening, and TPT on reducing TB incidence rates, and analysed the feasibility of these intervention combinations to achieve the goal of ending TB.

TB incidence in Shangrao City showed a consistent decline from 2008 to 2019, accelerated by strengthened control measures under national initiatives such as the Healthy China 2030 Blueprint and End TB Action Plan. This progress was supported by improved diagnostics, enhanced surveillance systems, and local interventions including active case finding implemented since 2008 (41, 42). However, while the reported incidence dropped sharply during the COVID-19 pandemic (2020–2022), SARIMA-adjusted projections suggest that without pandemic disruptions, TB incidence would have shown a gradual post-2020 increase. This upward trend may reflect rising recurrence rates among previously treated patients, compounded by the discontinuation of temporary COVID-19 containment measures (e.g., masking and reduced mobility) that inadvertently suppressed TB transmission (43). Without sustained intervention reinforcement, post-pandemic resurgence remains a concern, particularly given the aging population and potential gaps in TB-specific control measures to reduce TB incidence of TB.

Our study found that even with a 100% ACF rate, this measure alone cannot achieve the goal of eliminating TB incidence rates by 2035, consistent with the findings of Romain et al. (14). It was emphasised that without LTBI screening and TPT, regardless of the frequency of interventions, TB control strategy goals would not be met by 2035. Combining ACF with LTBI screening and TPT is expected to have a greater impact on the future TB burden. According to the WHO (44), approximately one-quarter of the global population has LTBI, posing a significant risk, as latent infections may progress to active TB under conditions of immune compromise or comorbidities. Recognising the importance of screening and TPT, which were identified as critical indicators of global TB control at the 2018 UN High-Level Meeting, is essential. China’s 2020 “Technical Specifications for Tuberculosis Prevention and Control Work” explicitly advocated for gradually implementing TPT for high-risk LTBI populations (45). Therefore, the importance of TB prevention and control in the older population cannot be overlooked. Analysis of TB incidence trends indicates that TB incidence rates among the older population show a gradual increase, possibly attributed to factors such as low sputum smear positivity rates, diagnostic challenges, immunosuppression, and the presence of multiple comorbidities affecting therapy efficacy (46, 47).

Moreover, our study found an increasing incidence of TB among individuals aged 65 years and above in the general population. By 2020, China’s population aged 60 years and above accounted for 18.7% of the total population, an 8.4% increase since 2000 (48). Wang et al. highlighted a three-fold higher TB incidence rate among the older than among the younger generations (49). Older individuals are not only more susceptible to new TB infections, but also face a higher risk of relapse. Therefore, the older population poses significant infection risks and public health challenges for TB prevention and control of TB. Despite this, current efforts to address and control TB among the older need to be strengthened (45), as the aging population in societies has significantly delayed the achievement of TB elimination goals. However, studies have shown that implementing TPT in the older is safe and feasible (15). Hence, future TB prevention and control efforts should pay special attention to the unique circumstances of the older population, including enhancing diagnostic accuracy, improving therapy adherence, and effectively managing relapse risk.

While intensifying active case-finding efforts, enhancing public health education and awareness among the population remains important. Our findings indicate that, in addition to ensuring high levels of active screening, boosting the proportion of the population receiving TPT is vital to achieving the aim of TB eradication. Simultaneously increasing both measures significantly accelerated PTB progression. Currently, compliance with TPT is generally low among patients with LTBI, often because of concerns regarding therapeutic efficacy and adverse reactions. Research indicates that awareness of LTBI among patients with LTBI directly influences their acceptance of TPT (50–52), with unfamiliarity with TPT content posing a major barrier.

Moreover, the current LTBI diagnosis lacks a unified gold standard, contributing to challenges in therapy acceptance owing to high false-positive rates. Interrupted therapy may lead to the development of drug resistance and further increase the risk of transmission of TB. However, studies by Bar-Meir et al. suggest that as patients’ knowledge of TB-related issues improves, their willingness to accept TPT significantly increases (53).

Furthermore, when comparing the five different TPT regimens, shorter therapy durations generally exhibited higher adherence, while regimens with shorter therapy durations, high compliance, and superior protection were more likely to achieve the goal of ending pulmonary TB. Future therapeutic drugs and regimens should further optimise the timing of TPT to ensure therapeutic efficacy and safety. Concurrently, improving the diagnostic accuracy and popularising health education among LTBI populations will help enhance the acceptability and effectiveness of TPT, thereby promoting effective TB control and eventual elimination. This study investigated the efficacy of five different preventive therapy regimens in reducing tuberculosis incidence based on their protective effects, adherence rates, and therapy durations. However, it does not currently address the variations in economic costs and adverse reactions among these regimens, which could be a promising direction for future research. Second, the data for this study were sourced from the national infectious disease reporting system, which may have suffered from underreporting and data gaps.

Additionally, relying solely on pulmonary tuberculosis incidence data to assess the feasibility of eradicating pulmonary tuberculosis may not provide a comprehensive view. Moreover, the interventions in this study did not specifically target high-risk populations but rather focused on the entire population across the two age groups.

## Conclusion

Based on our analysis of TB incidence trends in Shangrao City from 2008 to 2023, it is evident that while the incidence rate has shown a declining trend, achieving the goal of ending TB remains challenging under current control measures. Our findings suggest that relying solely on ACF will not suffice to meet the incidence reduction targets required to end TB. Therefore, it is essential to combine an ACF with TPT. Special attention must be paid to vulnerable populations,particularly the older, who face elevated risks of primary TB infection and disease relapse. Future TB control strategies should prioritise intensified health education tailored to this demographic, strict adherence to standardised treatment protocols, enhanced diagnostic techniques (e.g., rapid molecular testing), and critically strengthened follow-up monitoring for older patients to mitigate the risks of recurrence and reinfection. These efforts are likely crucial for achieving sustained TB control.

## Data Availability

The original contributions presented in the study are included in the article/Supplementary material, further inquiries can be directed to the corresponding author/s.
