# Correction: Feasibility of ending tuberculosis in Shangrao City through active intervention measures: a mathematical study

**DOI:** 10.3389/fpubh.2026.1785967

**Published:** 2026-02-06

**Authors:** Mingshu Xu, Yue He, Qiao Liu, Qiuping Chen, Zeyu Zhao, Zheng Xu, Chongfei Shu, Jun Xia, Yuyan Yang, Laurent Gavotte, Roger Frutos, Huiming Ye, Yanhua Su, Xiaolan Wang, Zhen Liu

**Affiliations:** 1 Shangrao Centre for Disease Control and Prevention, Shangrao City, China; 2 State Key Laboratory of Vaccines for Infectious Disease, Xiang An Biomedicine Laboratory, State Key Laboratory of Molecular Vaccinology and Molecular Diagnostics, National Innovation Platform for Industry-Education Intergration in Vaccine Research, School of Public Health, Xiamen University, Xiamen City, China; 3 CIRAD, URM 17, Intertryp, Montpellier, France; 4 Université de Montpellier, Montpellier, France; 5 Espace-Dev, Université de Montpellier, Montpellier, France; 6 Department of Laboratory Medicine, Fujian Key Clinical Specialty of Laboratory Medicine, Women and Children's Hospital, School of Medicine, Xiamen University, Xiamen City, China; 7 Shangrao People's Hospital, Shangrao City, China

**Keywords:** tuberculosis, dynamic model, end tuberculosis, active case finding, tuberculosis preventive therapy

The funder the Science and Technology Program of Jiangxi Provincial Health Commission, grant number: No. SKJP220236563-202410977, to Mingshu Xu was erroneously omitted.

In the published article, there was a mistake in the Funding statement. The funding statement was displayed as:

“This study was supported by Self-supporting Program of Guangzhou Laboratory (grant number: No. SRPG22-007) and Major Project of Guangzhou National Laboratory (grant number: No. GZNL2024A01004). The funder had no role in the study design, data collection and analysis, decision to publish, or manuscript preparation.”

The correct Funding statement is:

“The author(s) declared that financial support was received for this work and/or its publication. This study was supported by the Science and Technology Program of Jiangxi Provincial Health Commission (grant number: No. SKJP220236563-202410977, to Mingshu Xu), the Self-supporting Program of Guangzhou Laboratory (grant number: No. SRPG22-007), and Major Project of Guangzhou National Laboratory (grant number: No. GZNL2024A01004). The funder had no role in the study design, data collection and analysis, decision to publish, or manuscript preparation.”

The original version of this article has been updated.

