# Supplementary material for: Feasibility of ending tuberculosis in Shangrao City through active intervention measures: a mathematical study
Source: Front Public Health. 2025 Sep 10;13:1614339. doi: 10.3389/fpubh.2025.1614339 (PMC12457300; doi:10.3389/fpubh.2025.1614339)
Supplement: Supplementary file 1 [file Supplementary_file_1.docx]

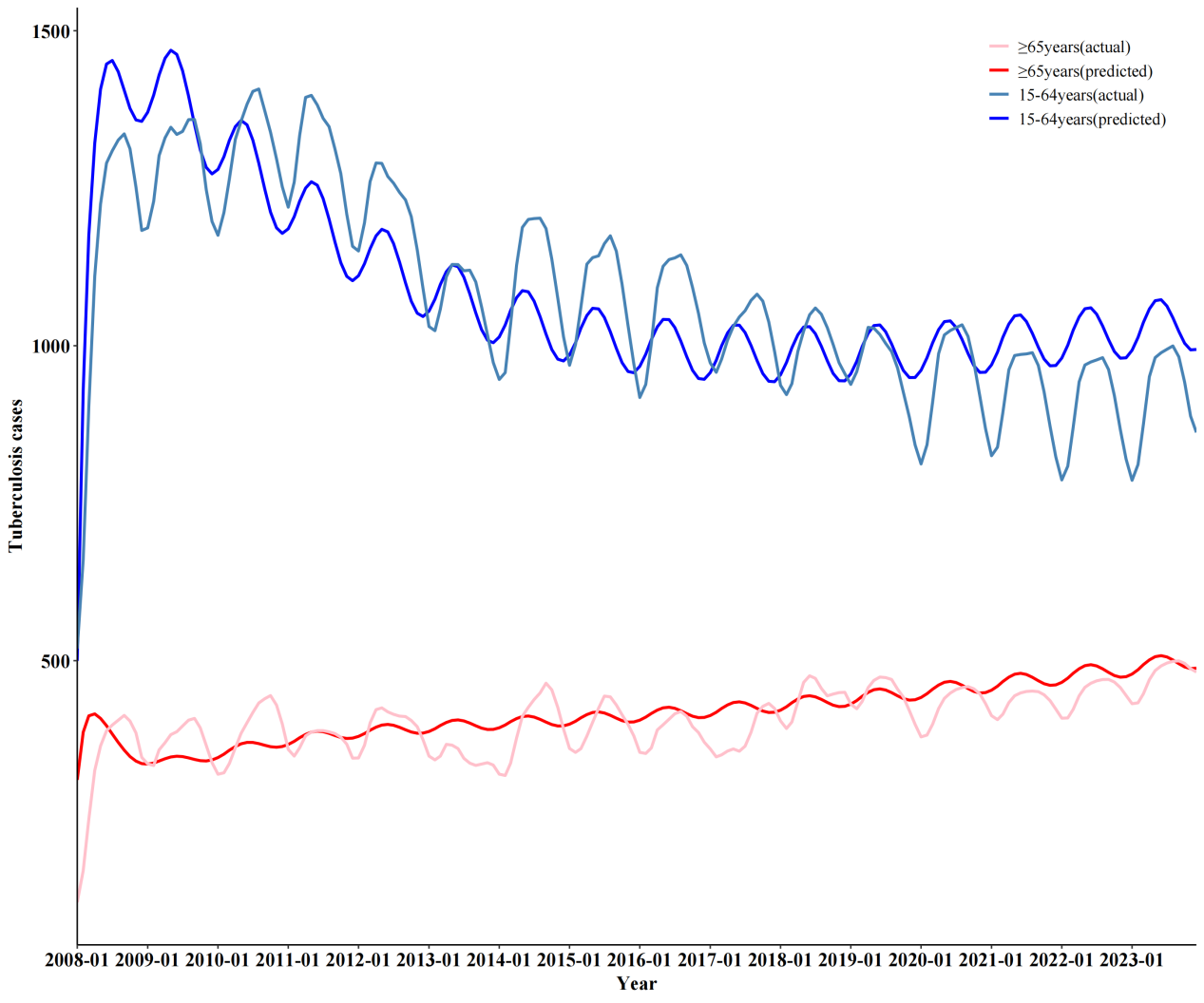


Figure S1 Fitting of *SEL_A_L_B_DR* Model to Tuberculosis Incidence in Shangrao City, 2008-2023


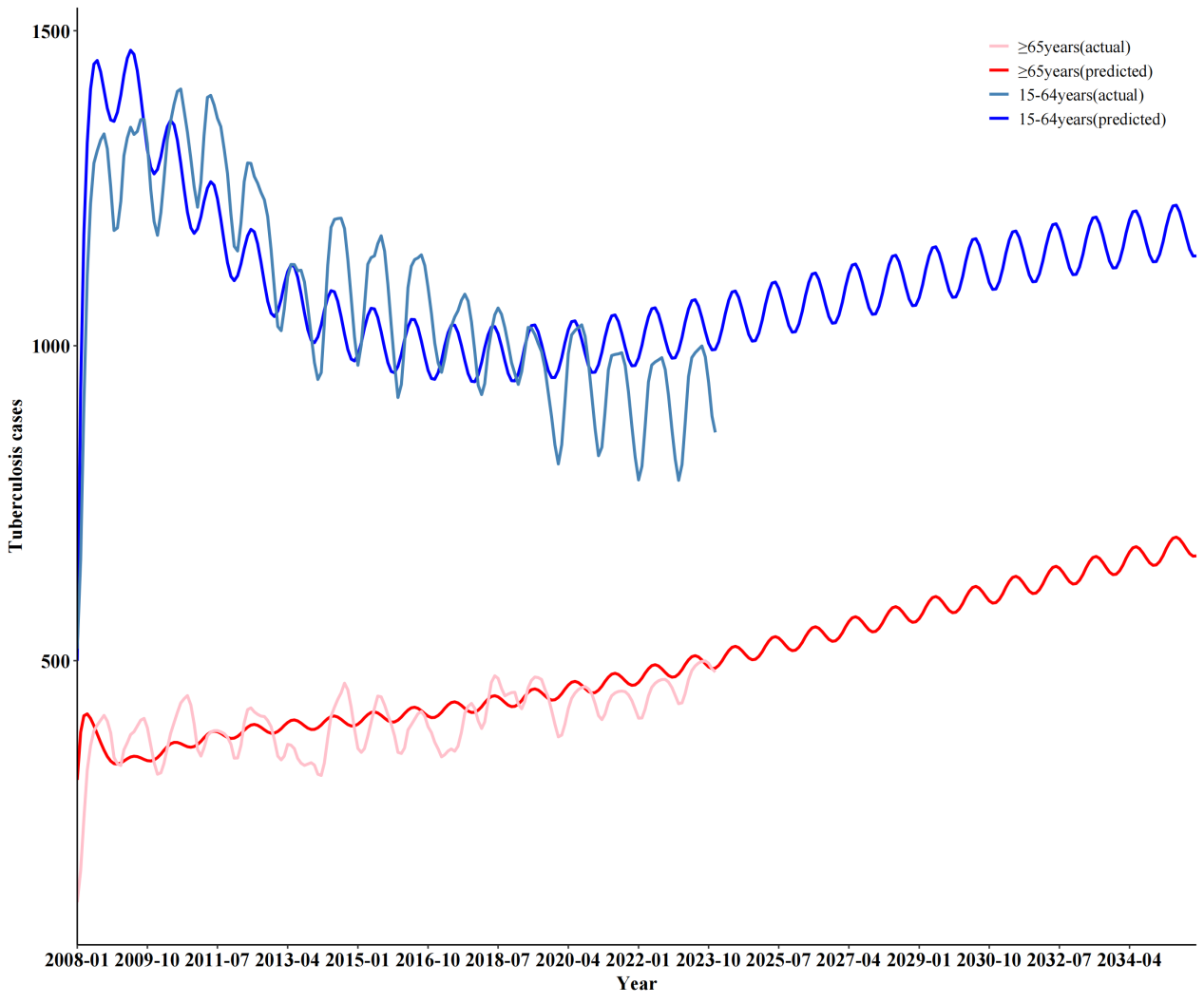


Figure S2 Fitting and Prediction of *SEL_A_L_B_DR* Model to Tuberculosis Incidence in Shangrao City, 2008-2035


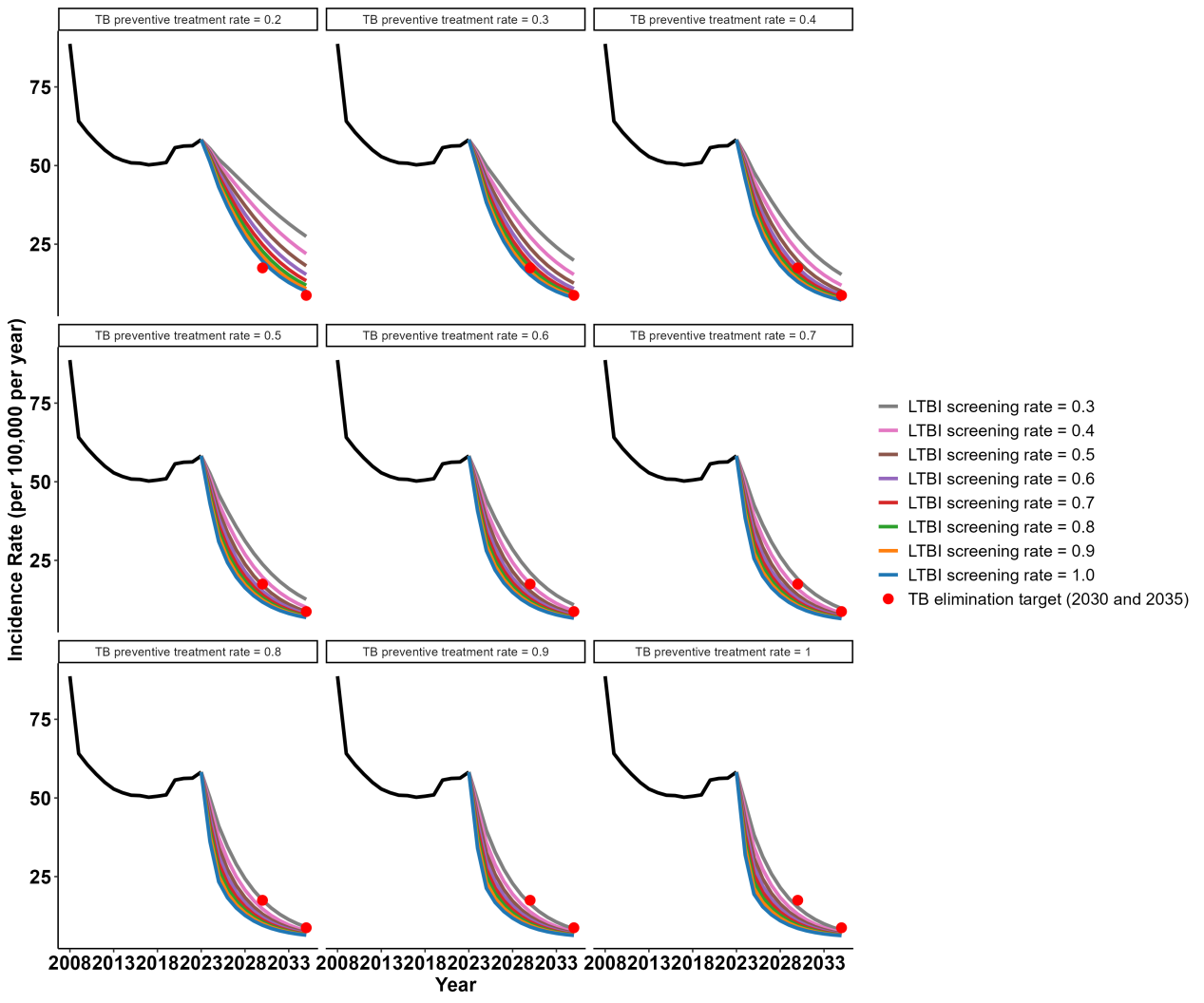


Figure S3 Prediction of incidence rate in Shangrao city based on Intervention B scheme 2


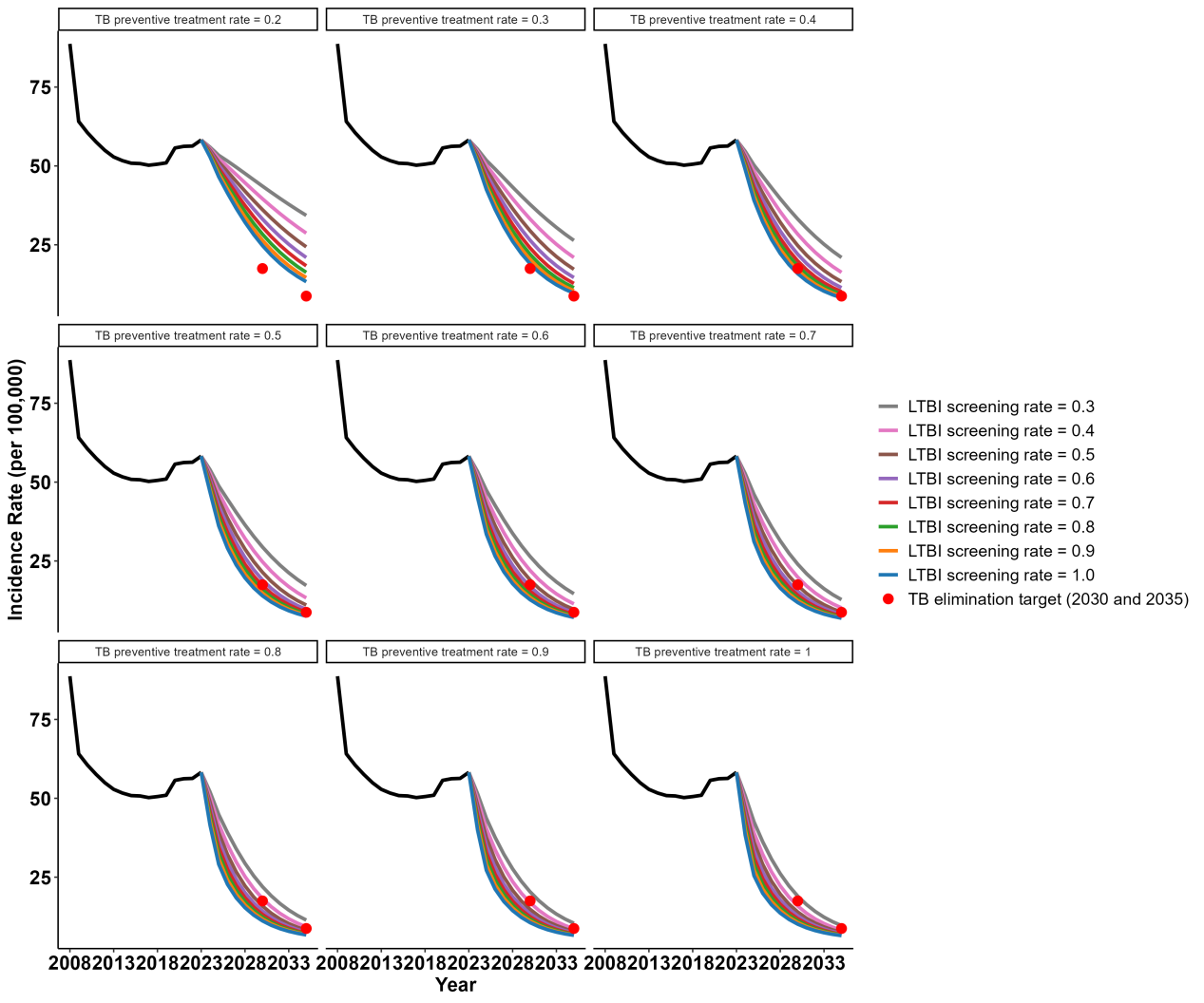


Figure S4 Prediction of incidence rate in Shangrao city based on Intervention B scheme 3


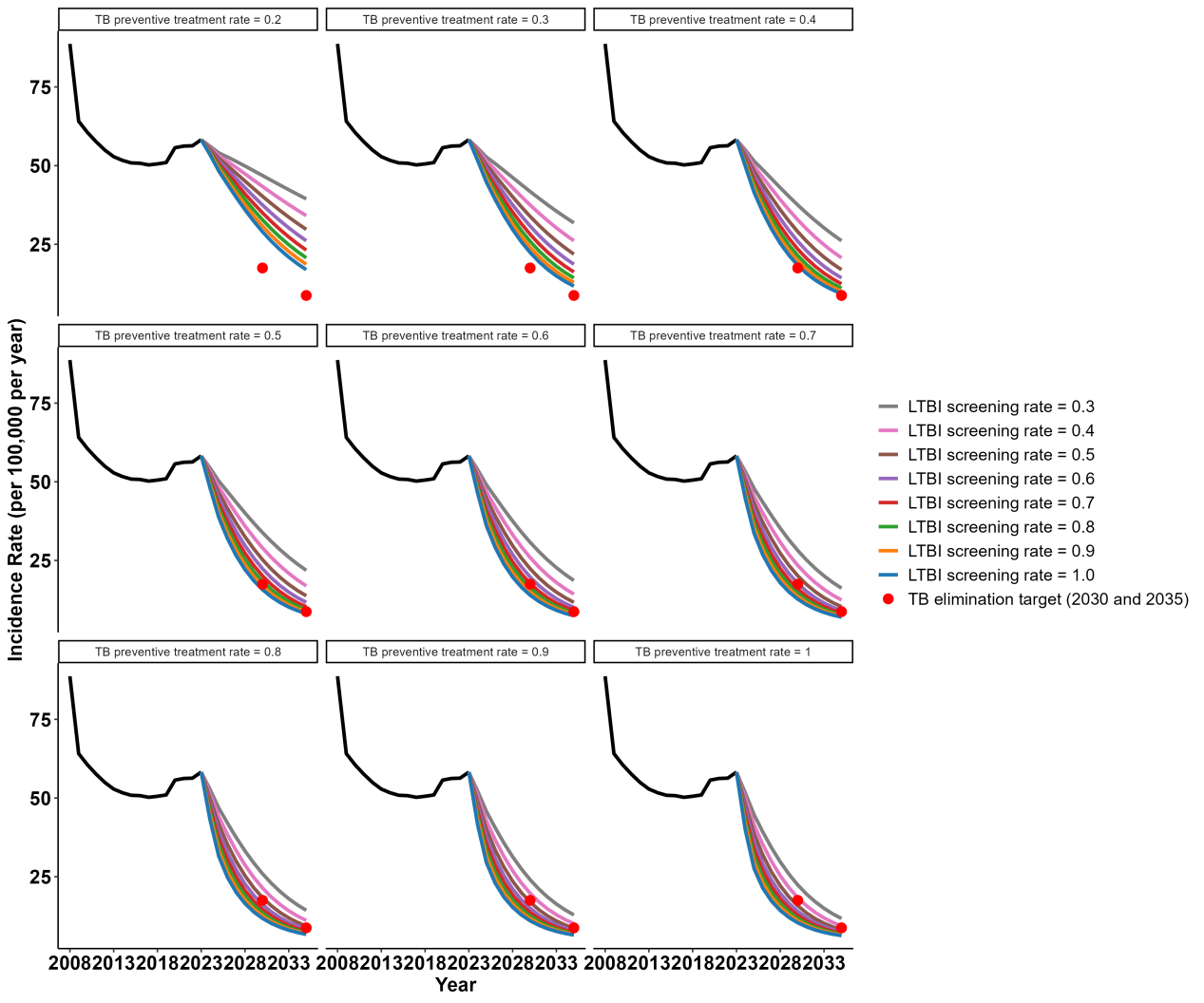


Figure S5 Prediction of incidence rate in Shangrao city based on Intervention B scheme 4


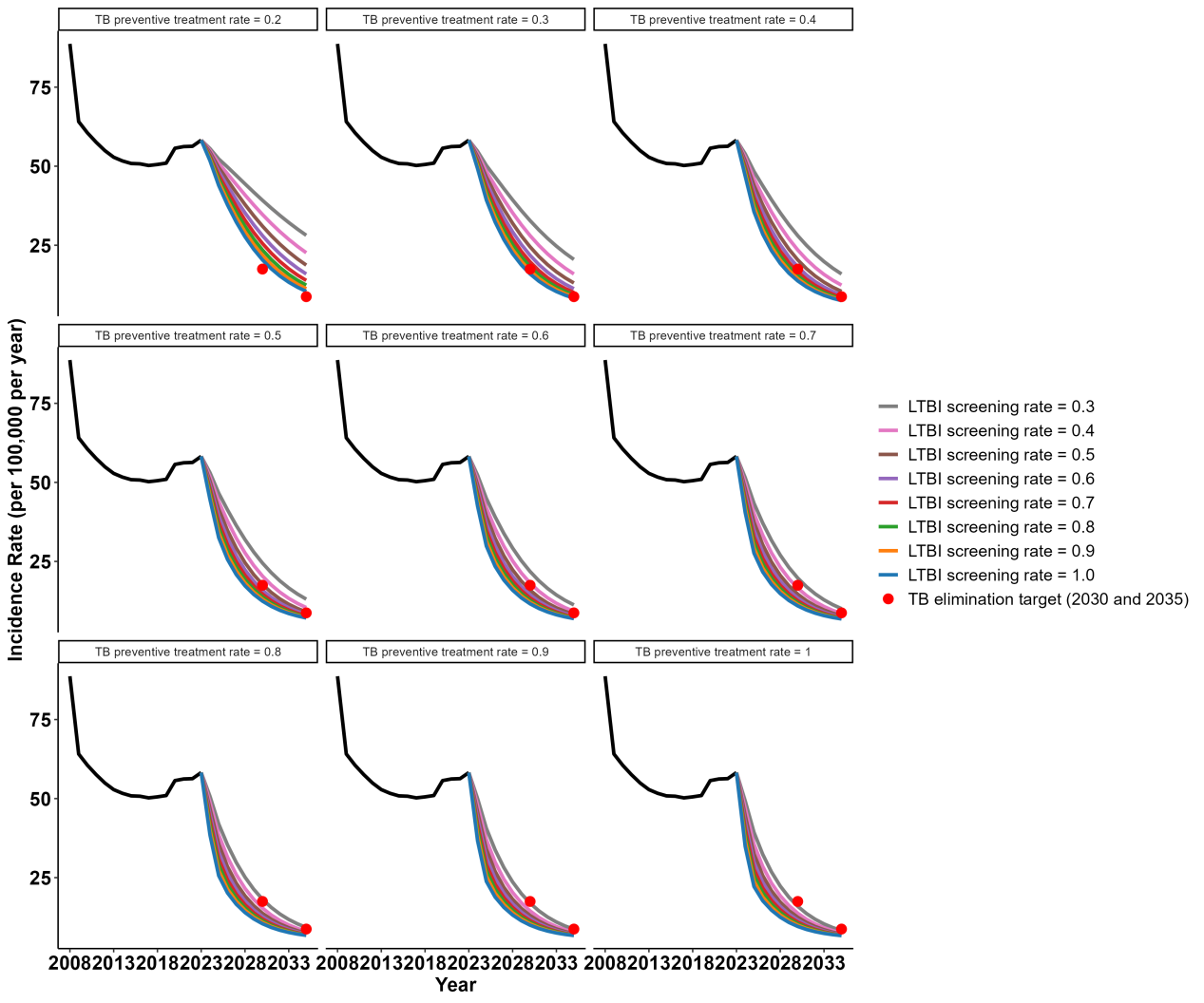


Figure S6 Prediction of incidence rate in Shangrao city based on Intervention B scheme 5


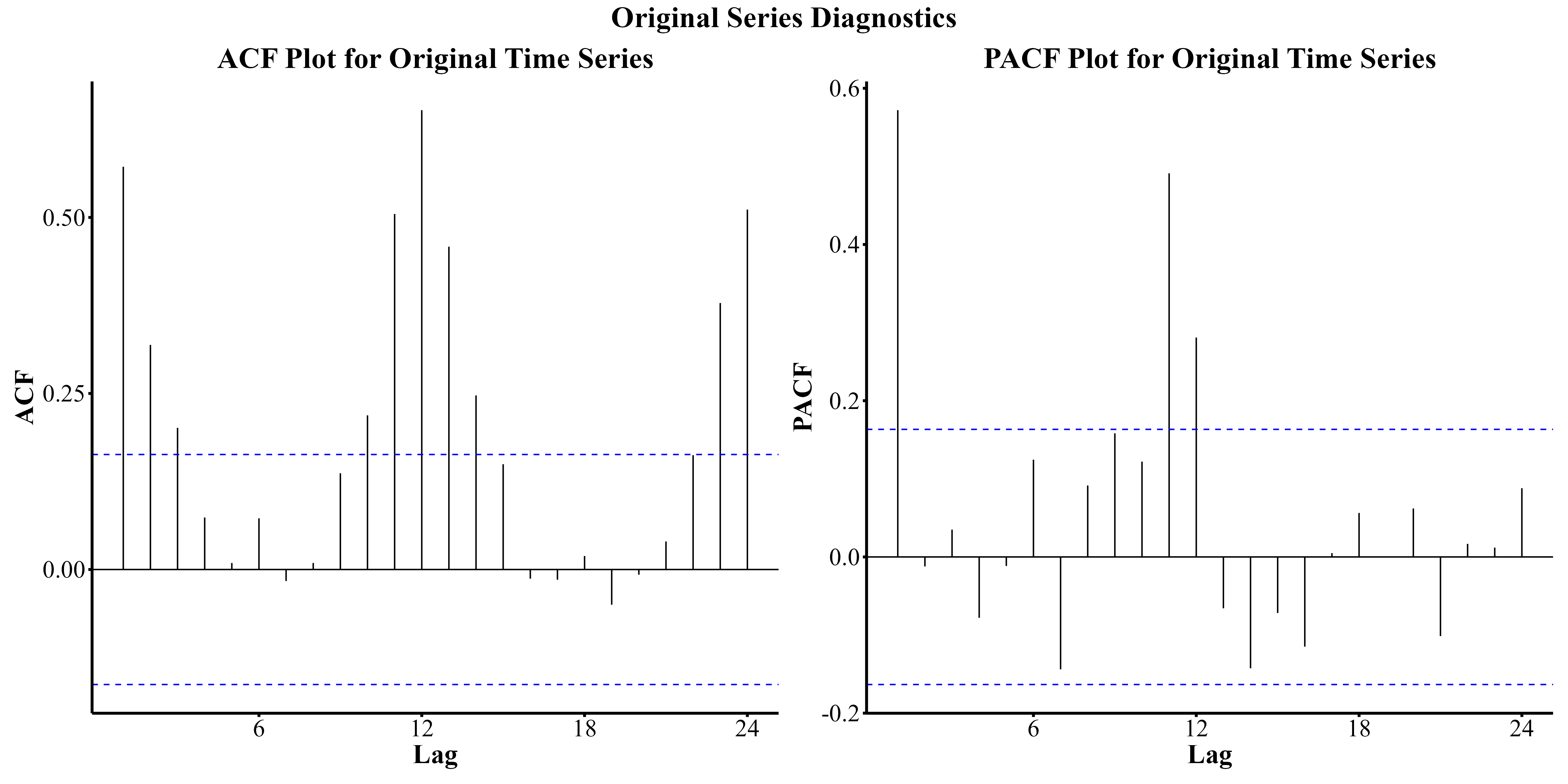


Figure S7 Autocorrelation function of the series of primary TB cases in the 15-65 age group


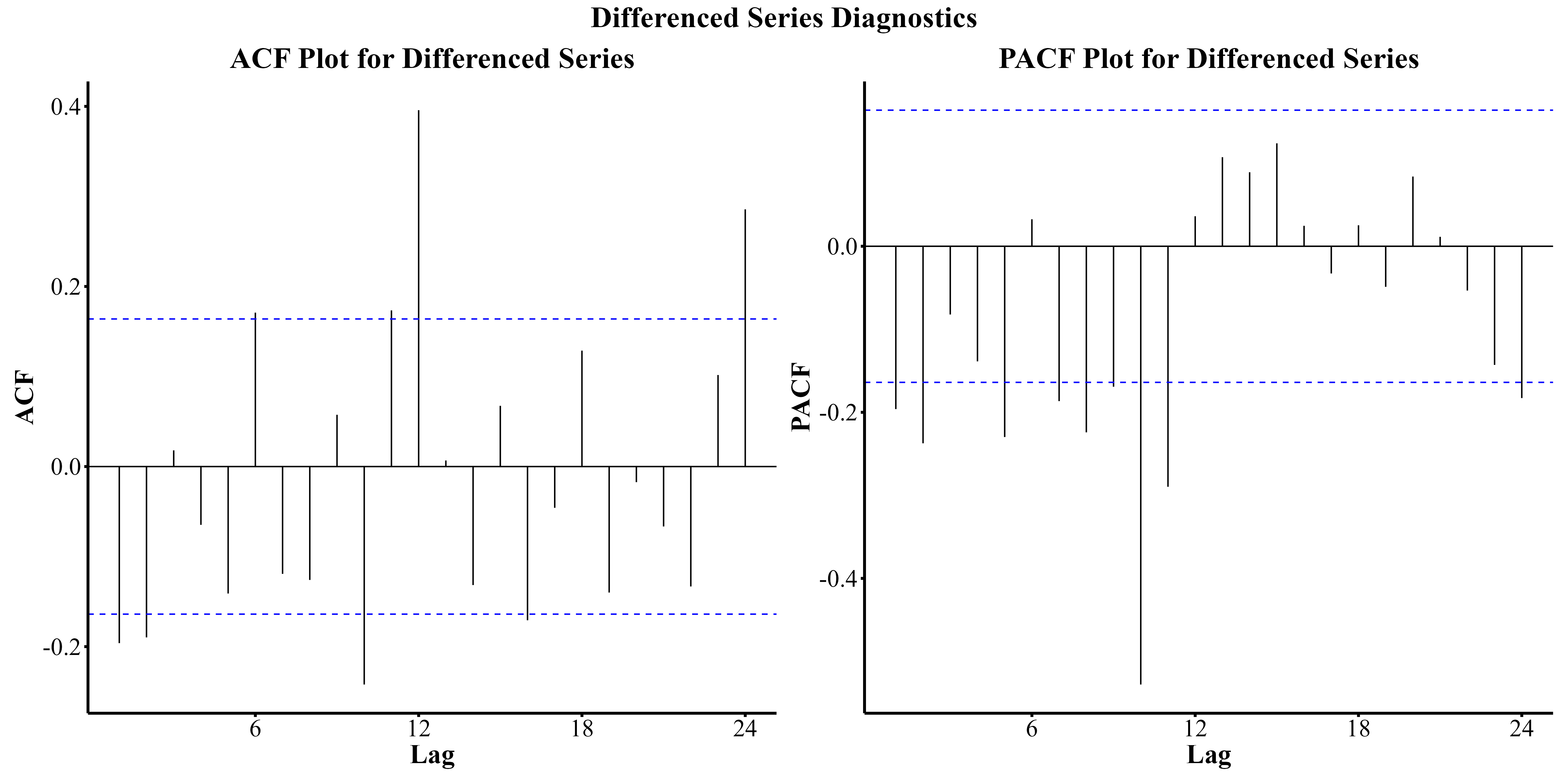


Figure S8 Stationarity Assessment of Differenced Tuberculosis Incidence in the 15-65 age group


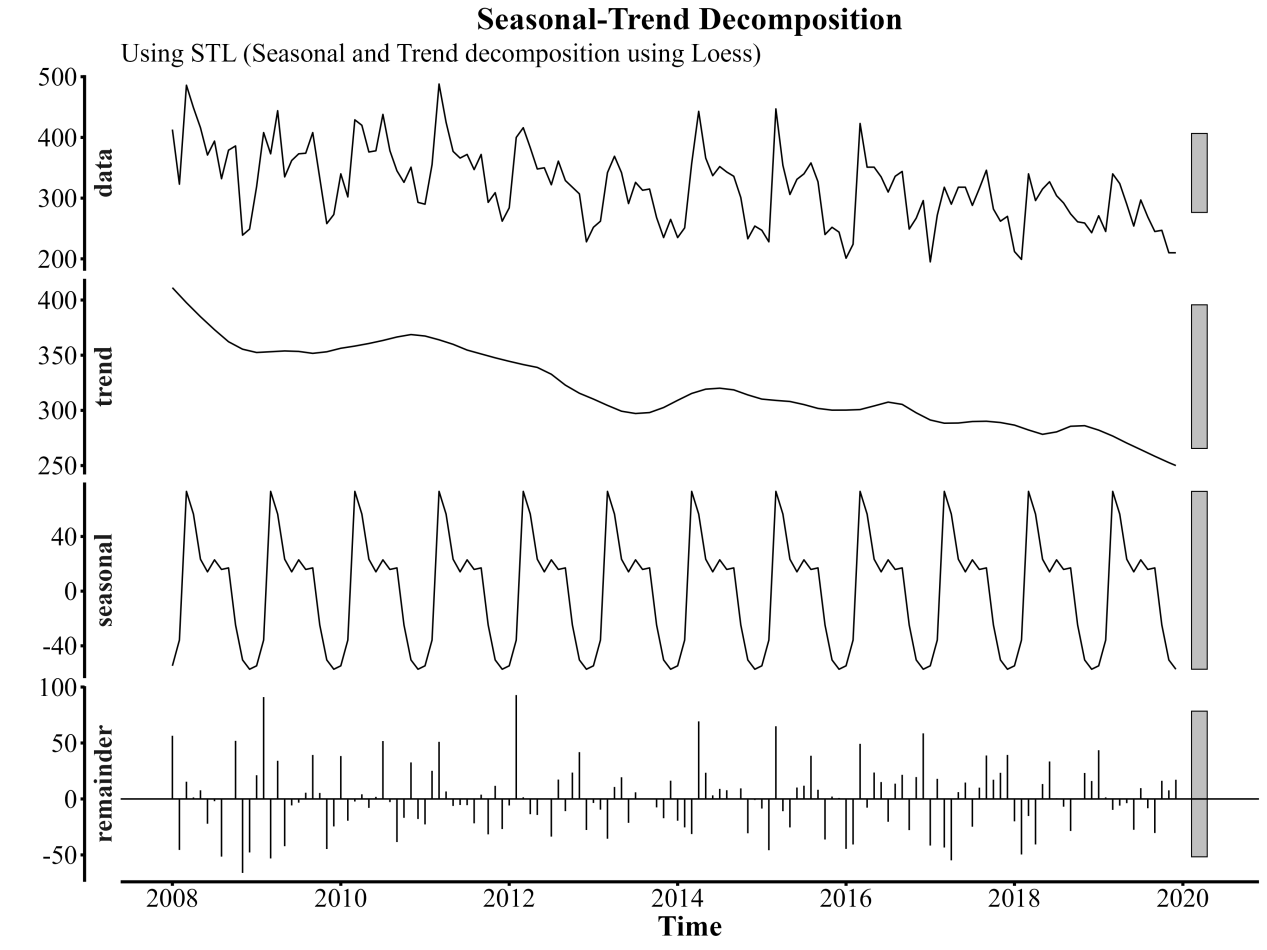


Figure S9 Seasonal-Trend Decomposition of Tuberculosis Epidemic Dynamics in the 15-65 age group


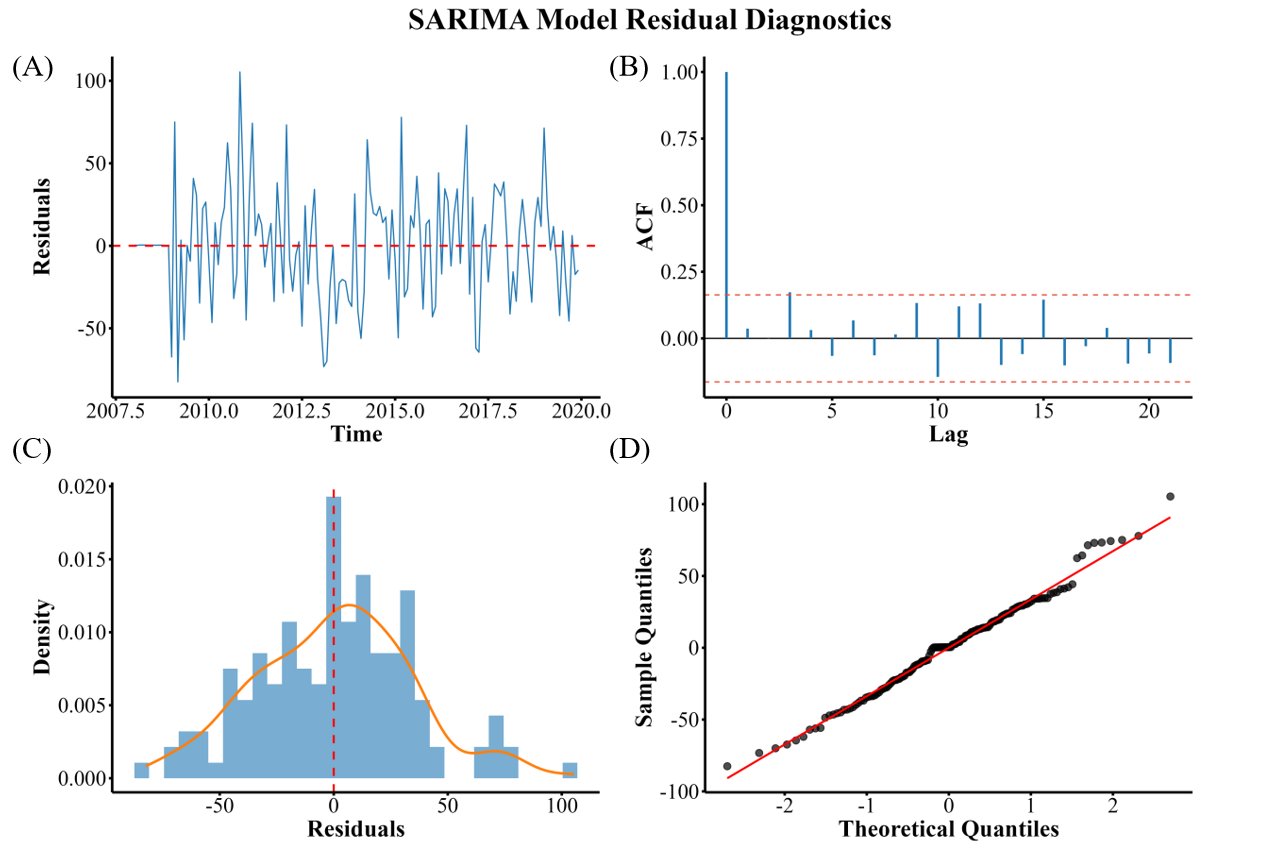


Figure S10 Residual Diagnostics for Tuberculosis Time Series Forecasting in the 15-65 age group

Diagnostic analyses reveal significant persistent seasonality in tuberculosis incidence patterns. The autocorrelation function (ACF) for the original series (Figure S7) exhibits a pronounced seasonal peak at lag 13 (ρ=0.65), indicating strong periodic fluctuations beyond annual cycles. Following first-order differencing, only 66.7% of autocorrelation coefficients fell within 95% confidence bounds (Figure S8), suggesting residual temporal dependencies requiring additional model refinement. The decomposed trend component (Figure S9) demonstrates a substantial monthly decline (β=-9.54 cases/month), signifying accelerated epidemic control during the observation period. Residual diagnostics (Figure S10) confirm adequate model specification, with normally distributed residuals (Shapiro-Wilk p=0.370) and no statistically significant autocorrelation (Ljung-Box p=0.069) at α=0.05 threshold, validating the modeling framework's core assumptions.


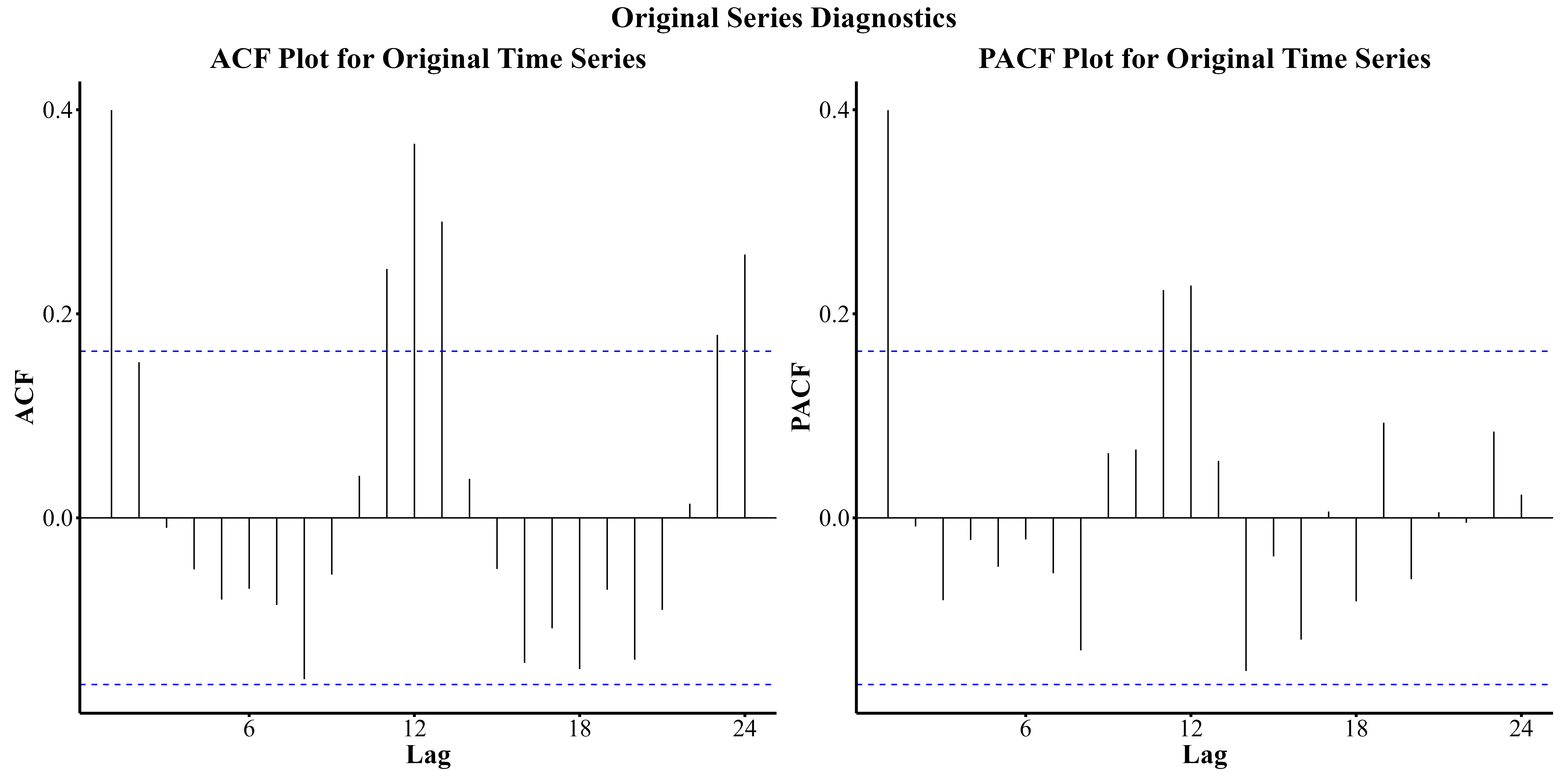


Figure S11 Autocorrelation function of the series of primary TB cases in the >65 age group


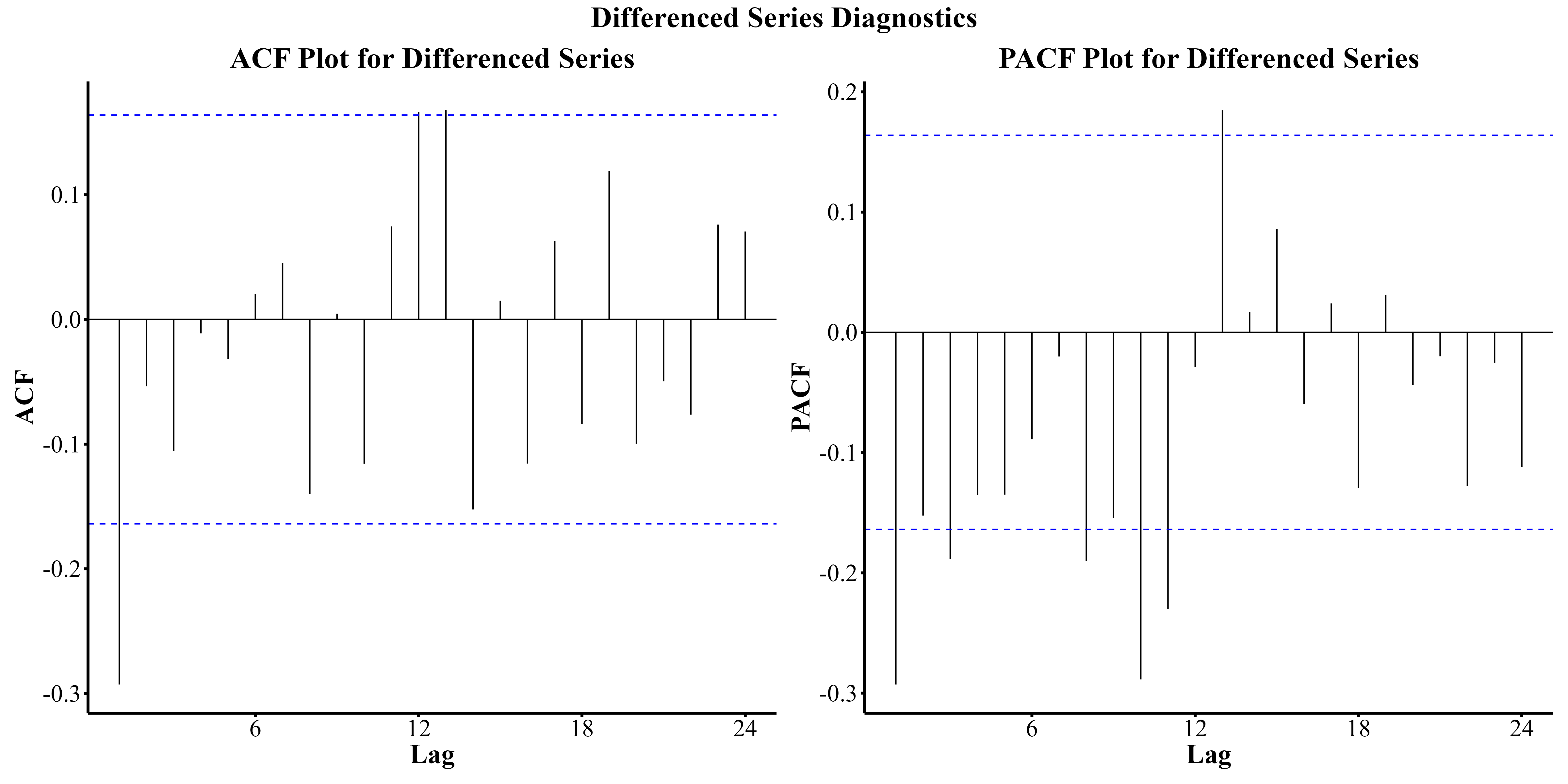


Figure S12 Stationarity Assessment of Differenced Tuberculosis Incidence in the >65 age group


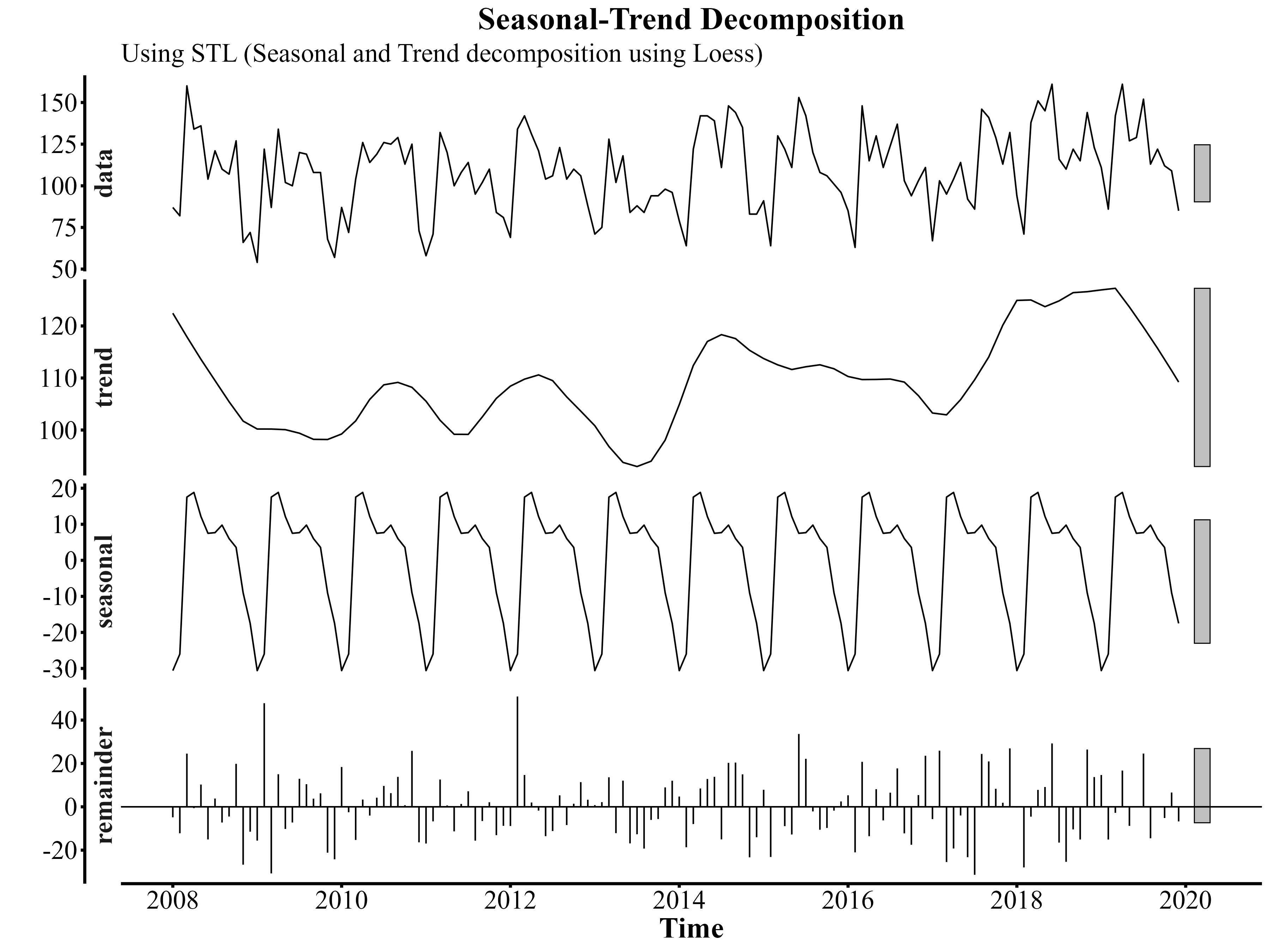


Figure S13 Seasonal-Trend Decomposition of Tuberculosis Epidemic Dynamics in the >65 age group


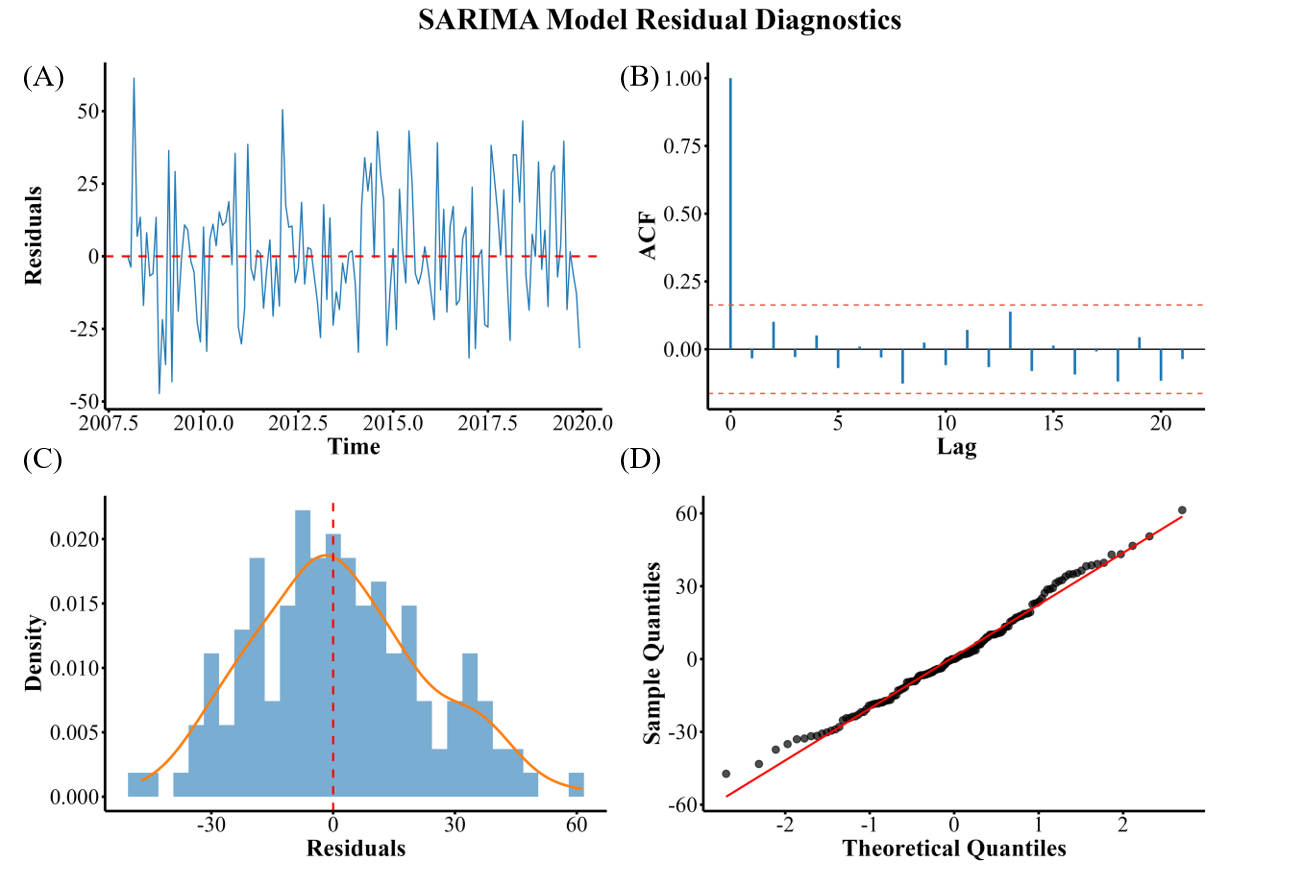


Figure S14 Residual Diagnostics for Tuberculosis Time Series Forecasting in the >65 age group

Diagnostic assessments identify persistent short-term cycles in tuberculosis transmission dynamics. The autocorrelation function (Figure S11) reveals significant short-term periodicity at lag 2 (ρ=0.40), indicating bi-monthly infection patterns distinct from annual seasonality. Post-differencing diagnostics (Figure S12) demonstrate effective stationarity transformation, with 87.5% of autocorrelations within 95% confidence bounds, confirming adequate differencing order specification. Trend decomposition analysis (Figure S13) identifies a concerning upward trajectory (β=+1.47 cases/month), signaling potential resurgence in disease burden. Final residual validation (Figure S14) substantiates model integrity, showing normally distributed errors (Shapiro-Wilk p=0.380) and statistically insignificant autocorrelation (Ljung-Box p=0.584) at α=0.05 level, fulfilling all modeling prerequisites.

Table S1 Model parameters

| Parameter | Description | Unit | Value | Source |
| --- | --- | --- | --- | --- |
| *β* | Transmission rate | person^-1^*month^-1^ | 8.39e-07  4.46e-07  2.20e-20  2.55e-19 | Fit |
| *m* | Proportion of self-clearance | 1 | 0.41 | Reference[1] |
| *θ* | Rate of self-clearance | month^-1^ | 1/13.75 | Reference[1] |
| *ε* | Rate of early progression | month^-1^ | 0.024/12;  0.045/12; | Reference[2, 3] |
| *α* | Rate of immune stabilization | month^-1^ | 0.454/12 | Reference[2] |
| *ω* | Rate of late progression | month^-1^ | 0.0018/12;  0.0027/12; | Reference[3, 4] |
| *φ* | Rate of spontaneous recovery | month^-1^ | 0.20/12;  0.14/12; | Reference[5, 6] |
| *δ* | Proportion of passive case detection | 1 | 0.74 | Reported data |
| *υ* | Rate of diagnosis of TB patients, i.e., the reciprocal of diagnosis delays | month^-1^ | 1/6.13~1/6.10* | Reported data |
| *λ* | Proportion of treatment success | 1 | 0.79~0.99* | Reported data |
| *γ* | Treatment recovery period for TB patients, i.e. the reciprocal of the patient's treatment duration | month^-1^ | 0.09~1.43* | Reported data |
| *r* | Proportion of relapse | 1 | 0.005 | Reference[7] |
| *η* | Rate of relapse | month^-1^ | 0.01/12;  0.1025/12; | Reference[6] |
| *μ_1_* | Fatality rate in patients with pathogenetically positive TB | month^-1^ | 0.13/12;  0.19/12; | Reference[5, 6] |
| *μ_2_* | Fatality rate in patients with pathogenetically negative TB | month^-1^ | 0.13/12;  0.19/12; | Reference[5, 6] |
| *χ* | the sensitivity of active screening | 1 | 0.90 | Reference[8, 9] |
| *τ* | LTBI diagnostic sensitivity | 1 | 0.75 | Reference  [10] |

Parameters with two values represent the two modeled age groups.

*The parameter values are determined based on the annual reported data of the local area.

[1] Verrall, A. J.; Alisjahbana, B.; Apriani, L.; Novianty, N.; Nurani, A. C.; van Laarhoven, A.; Ussher, J. E.; Indrati, A.; Ruslami, R.; Netea, M. G. Early clearance of Mycobacterium tuberculosis: the INFECT case contact cohort study in Indonesia. *The Journal of infectious diseases* **2020**, *221* (8), 1351-1360.

[2] Diel, R.; Loddenkemper, R.; Niemann, S.; Meywald-Walter, K.; Nienhaus, A. Negative and positive predictive value of a whole-blood interferon-γ release assay for developing active tuberculosis: an update. *American journal of respiratory and critical care medicine* **2011**, *183* (1), 88-95.

[3] Shrestha, S.; Kendall, E. A.; Chang, R.; Joseph, R.; Kasaie, P.; Gillini, L.; Fojo, A. T.; Campbell, M.; Arinaminpathy, N.; Dowdy, D. W. Achieving a “step change” in the tuberculosis epidemic through comprehensive community-wide intervention: a model-based analysis. *BMC medicine* **2021**, *19*, 1-15.

[4] Colijn, C.; Cohen, T.; Murray, M. Mathematical models of tuberculosis: accomplishments and future challenges. In *BIOMAT 2006*, World Scientific, 2007; pp 123-148.

[5] Tiemersma, E. W.; van der Werf, M. J.; Borgdorff, M. W.; Williams, B. G.; Nagelkerke, N. J. Natural history of tuberculosis: duration and fatality of untreated pulmonary tuberculosis in HIV negative patients: a systematic review. *PloS one* **2011**, *6* (4), e17601.

[6] Harris, R. C.; Sumner, T.; Knight, G. M.; Evans, T.; Cardenas, V.; Chen, C.; White, R. G. Age-targeted tuberculosis vaccination in China and implications for vaccine development: a modelling study. *The Lancet Global Health* **2019**, *7* (2), e209-e218.

[7] Harris, R. C.; Sumner, T.; Knight, G. M.; Evans, T.; Cardenas, V.; Chen, C.; White, R. G. Age-targeted tuberculosis vaccination in China and implications for vaccine development: a modelling study. *Lancet Glob Health* **2019**, *7* (2), e209-e218. DOI: 10.1016/s2214-109x(18)30452-2 From NLM.

[8] Steingart, K. R.; Schiller, I.; Horne, D. J.; Pai, M.; Boehme, C. C.; Dendukuri, N. Xpert® MTB/RIF assay for pulmonary tuberculosis and rifampicin resistance in adults. *Cochrane Database Syst Rev* **2014**, *2014* (1), Cd009593. DOI: 10.1002/14651858.CD009593.pub3 From NLM.

[9] WHO Guidelines Approved by the Guidelines Review Committee. In *Systematic Screening for Active Tuberculosis: Principles and Recommendations*, World Health Organization

Copyright © World Health Organization 2013., 2013.

[10] Wang LiXia, W. L.; Zhang Hui, Z. H.; Ruan YunZhou, R. Y.; Chin, D.; Xia YinYin, X. Y.; Cheng ShiMing, C. S.; Chen MingTing, C. M.; Zhao YanLin, Z. Y.; Jiang ShiWen, J. S.; Du Xin, D. X. Tuberculosis prevalence in China, 1990-2010; a longitudinal analysis of national survey data. **2014**.
